# Action Modulates the Conscious Reasoning Process of Moral Judgment: Evidence From Behavior and Neurophysiology

**DOI:** 10.3389/fnbeh.2020.577252

**Published:** 2021-01-06

**Authors:** Yue Leng, Jili Zhang, Yanan Zhangyu, Xiaoyuan Yang

**Affiliations:** ^1^School of Biomedical Science and Medical Engineering, Southeast University, Nanjing, China; ^2^Research Center for Learning Science, Southeast University, Nanjing, China; ^3^Key Laboratory of Child Development and Learning Science (Ministry of Education), Southeast University, Nanjing, China

**Keywords:** moral dilemma, allowing harm, event-related potentials, N450, LPP, doing harm

## Abstract

Moral judgment can be highly affected by the action and intention factors on a behavior level. Previous neuroimaging studies have demonstrated that the intention factor can modulate both the affective and cognitive processing of moral judgment. The present event-related potentials (ERP) study examined how the action factor modulated the neural dynamics of moral judgment under a newly developed moral dilemma paradigm including three different conditions: harm caused by action (i.e., doing harm), harm caused by omission (i.e., allowing harm), and no harm. Behavior data showed that participants preferred utilitarian judgments and spent less time on the allowing harm condition than for the doing harm condition. ERP results revealed that, compared with the doing harm and no harm dilemmas, the allowing harm dilemmas elicited an enhanced N450 response associated with cognitive control and/or cognitive effort processes, but attenuated a late positive potentials (LPP) response associated with top-down control of attention and cognitive “rational” control processes. Such LPP amplitude differences were positively correlated with the C-score of the moral competence test which indexed the cognitive aspect of moral judgment competency. These findings suggested that people have a strong omission bias, and such an action factor modulates the conscious reasoning process during moral judgment, including the cognitive control and/or cognitive effort, and attentional allocation processes.

## Introduction

During the second half of twentieth century, the argument on how people make moral decisions mainly focused on two schools of thoughts in moral psychology. One following Kant's categorical imperative, holding that people making moral decisions in controlled, rational, and reflective ways, engendered rational models of moral decision-making (Colby and Kohlberg, 1987). The other, affected by Hume's critique of reason, insisting that people making moral decisions in automatic, emotional, and intuitive ways, led to emotional and intuitive models of moral decision-making (Haidt, 2001). Until neuroimaging techniques were introduced into moral psychology, the dual processes theory was proposed to explain the psychological and neural mechanisms of moral judgment on moral dilemmas (Greene et al., 2001, 2004, 2008). Recently, evolutionary psychology elaborated on the dual-processing model that people making moral decisions in a moral dilemma included both an automatic “emotional-intuitive” process and a controlled “rational-reflective” process. The former process was guided by primitive brain systems eliciting emotional responses; the latter process was guided by later evolved brain systems, yielding reasoned responses. Importantly, it even emphasized that different processes of moral judgment may be triggered by different types of moral dilemmas (Denton and Krebs, 2016).

Over the past 20 years, moral dilemmas have been more frequently used to investigate psychological and neural mechanisms of moral judgment. When facing various types of moral dilemmas, people may select diverse moral principles to make judgments, for example, Utilitarianism or Deontology, which are two opposing classical moral principles in traditional moral philosophy. Utilitarianism, proposed by Bentham, refers to maximizing benefits and minimizing costs (Schneider, 1981). Deontology, proposed by Kant, is the normative ethical theory that the morality of an action should be based on whether that action itself is right or wrong under a series of rules, rather than based on the consequences of the action (McCain, 1991). Indeed, by using multiple moral dilemma tasks, functional magnetic resonance imaging (fMRI) studies, as well as brain lesion studies, have differentiated individuals' brain responses when using Utilitarianism thinking from when using Deontological thinking to make moral judgements. Emotional brain areas such as the ventromedial prefrontal cortex (VMPFC) and amygdala may lead to deontological judgments, and cognitive brain areas such as the dorsolateral prefrontal cortex (DLPFC) and anterior cingulate cortex (ACC) may lead to utilitarian judgments (Greene et al., 2001; Koenigs et al., 2007; Shenhav and Greene, 2014; Chen et al., 2016). Additionally, Greene and colleagues emphasized the role of cognitive control afforded by the DLPFC and ACC in overriding the emotional processing which recruited the medial prefrontal cortex (MPFC), posterior cingulate cortex (PCC), and superior temporal sulcus (STS) (Greene et al., 2004; Cushman et al., 2010).

Furthermore, there are several formulations of Deontology, e.g., the Doctrine of Doing and Allowing (DDA) (Quinn, 1989) and the Doctrine of the Double Effect (DDE) (Foot, 1985). The DDA states that harm caused by action is worse than harm caused by omission (Baron and Ritov, 2004). In contrast, the DDE refers to harm intended as the means to a goal being worse than harm foresaw as the side effect of a goal (Mcintyre, 2008). In moral psychology, the DDA and DDE correspond to the action and intention principles, respectively. To be noted, action (Ritov and Baron, 1990), intention (Leslie et al., 2006), and contact (Greene et al., 2001) are regarded as three common principles that guide moral judgments on moral dilemma. To investigate how the action and intention factors affect psychological and neural mechanisms of moral judgments on moral dilemmas, several studies provided robust behavior and neuroimaging evidence. An earlier behavior study using the moral sense test (MST) pointed out that the action principle was available to the conscious reasoning system for moral judgment, whereas the intention principle is better characterized by the intuition system for moral judgment (Cushman et al., 2006). Simultaneously, Schaich and her colleagues developed a set of moral scenarios to describe variations in moral dilemmas (i.e., action, consequence, and intention), and conducted an fMRI study. They found that action scenarios, in relative to omission scenarios, elicited stronger activations in brain areas related to conscious processes, e.g., the regulation of negative emotion (i.e., the right middle frontal gyrus or rostral DLPFC). These same scenarios, however, evoked weaker activations in brain areas associated with emotional processing, such as the orbitofrontal cortex (OFC) and temporal pole (TP) (Schaich Borg et al., 2006).

With respect to neural dynamics of moral judgments on moral dilemmas, a few event-related potentials (ERP) studies were conducted using the same standardized set of moral dilemmas under the framework of the DDE, including instrumental dilemmas in which killing one person was an intended way to save other people, and incidental dilemmas wherein killing one person was a foreseen but unintended consequence. The authors consistently proposed that brain activities in moral judgment can be divided into an early immediate affective processing stage and a late controlled cognitive processing stage, and found how temporal dynamics of moral judgment were affected by the intention principle. During the early stage, the frontal P260 reflecting an immediate affective appraisal of the available options might be associated with neural activities in the orbital/medial prefrontal regions. It was more positive for the instrumental dilemmas than for the incidental dilemmas. For the late stage, the parietal slow waves in the time window of 300–450, 450–600, and 600–750 ms, indexing attentional resource distribution and working memory load, was more positive for the incidental dilemmas than for the instrumental dilemmas (Sarlo et al., 2012; Pletti et al., 2015). In addition, given that the involvement of cognitive control in moral judgments on moral dilemmas (Greene et al., 2004; Cushman et al., 2010), some cognitive-control-related ERP potentials would be observed during brain electrical responses to a moral dilemma resolution. To our knowledge, the frontocentral N450, also occasionally called the medial frontal negativity (West, 2003; Chen et al., 2011; West and Bailey, 2012), is a negative ERP deflection localized in the anterior cingulate cortex (ACC) or lateral prefrontal cortex, peaking ~450 ms after stimulus onset, reflecting the cognitive control processes and/or cognitive effort during conflict processing (Botvinick et al., 2004; Larson et al., 2014; Chmielewski and Beste, 2017), and is very likely to be modulated by variations in cognitive control (Stock et al., 2016).

However, whether the time course of neural responses to moral judgments on moral dilemmas guided by other classical moral principles (e.g., the DDA/action principle) resembles the pattern above, and how the DDA/action principle modulates the temporal dynamics of moral judgment on a moral dilemma, are unknown. By using ERP, the present study aimed at investigating the cognitive-emotional interplay in moral judgment with a set of dilemmas inspired by the DDA, which is a principle of deontology. We designed an experimental task in which participants were asked to read a scenario followed by three proposed protagonist's resolutions, i.e., harm caused by action (doing harm), harm caused by omission (allowing harm), and no harm, and then make a decision on whether they agreed or disagreed with the protagonist's resolution. We examined the behavioral and ERP responses of people facing moral dilemmas under three conditions. Under the guidance of the DDA, doing harm is worse than allowing harm, therefore in the condition of doing harm, emotional evaluation would overcome the cognitive processes leading to deontological responses; on the contrary, in the condition of allowing harm, cognitive processes would defeat the emotional evaluation, resulting in utilitarian responses. We hypothesized that, when judging the resolution of moral dilemmas involving harm caused by action, fewer participants would say “yes” to doing harm, and the conscious reasoning system for moral judgment would be less recruited. In contrast, when judging the resolution of moral dilemmas involving harm caused by omission, more participants would say “yes” to allowing harm, and the conscious reasoning system would be more engaged. More critically, such rational engagement would be reflected in the late controlled processing stages indexed by the late ERP components, e.g., N450 or the parietal slow waves.

## Methods

### Participants

Seventeen undergraduate or graduate students (11 females) were recruited from the Intranet of Southeast University. Participants were aged 21–29 years (M = 23.76, SD = 2.73), were right-handed, had no history of neurological problems, and had normal or corrected-to-normal vision. They were paid 50 RMB for their participation and gave their informed consent, which was approved by the Ethics Committee of Affiliated Zhongda Hospital, Southeast University, China (2016ZDSYLL002.0).

### Stimulus Materials

We newly developed 180 experimental moral and 12 filler non-moral dilemmas. All the moral dilemmas were limited to the issue of death rather than other moral issues. Each dilemma was composed of the scenario and the protagonist's resolution. On the basis of the DDA (Howardsnyder, 2016), there were 60 experimental moral scenarios with three types of resolution: harm caused by action (doing harm), harm caused by omission (allowing harm), and no harm. For each non-moral scenario, there was only one type of protagonist's resolution. Both the scenario and the protagonist's resolution were presented as text on a computer screen. To highlight the contrast among different protagonist's resolutions, the scenario was displayed as a paragraph composed of four sentences indicating background, reason, consequence, and linking up components. Then, the protagonist's resolution was displayed in a verb-object phrase. The mean number of words for both the scenarios and resolutions in Chinese were fully balanced across the three types of moral dilemmas and non-moral dilemmas. The filler non-moral dilemma condition was not analyzed and will not be discussed further here. The Chinese and English translated version of all the experimental and filler dilemmas can be downloaded as Supplementary Material. Some examples of experimental dilemmas are listed in Table 1.

**Table 1:** One example of the experimental moral dilemmas for three resolution types. The standardized structure of scenario includes background, reason, consequence, and linking up components.

**Resolution type**	**Scenario**	**Protagonist's action**	**Object of the protagonist's action**
Doing harm	Background: *An emergency physician additionally receives five slightly injured patients when giving a blood transfusion to a critically injured patient. If the patients with slight injury do not receive blood transfusion in time, their conditions will deteriorate and they will die, but the blood bank almost runs out of blood at this time*. Reason: *In order to save more lives, the doctors take some actions on the patient with serious injury*. Consequence: *Five patients with slight injury are saved, but the patient with serious injury dies*. Linking up components: *the actions taken by the doctor are…*	Cutting off	Blood transfusion
Allowing harm	Background: *An emergency physician additionally receives five slightly injured patients when giving a blood transfusion to a critically injured patient. If the patients with slight injury are not treated in time in time, their conditions will deteriorate and they will die, but the manpower is in shortage at this time*. Reason: *In order to save more lives, the doctors take some actions on the patient with serious injury*. Consequence: *Five patients with slight injury are saved, but the patient with serious injury dies*. Linking up components: *the actions taken by the doctor are…*	Giving up	Treatment
No harm	Background: *An emergency physician additionally receives five slightly injured patients when giving a blood transfusion to a critically injured patients. If the patients with slight injury are not treated in time, their conditions will deteriorate and they will die, and colleagues of the doctor are available at this time*. Reason: *In order to save more lives, the doctors take some actions on the patient with serious injury*. Consequence: *Five patients with slight injury are saved, but the patient with serious injury dies*. Linking up components: *the actions taken by the doctor are…*	Asking for	Assistance

Prior to the formal experiment, a pilot experiment was conducted, in which 44 undergraduate or graduate students (24 females, M = 25.07, SD = 2.29) were recruited from the Intranet of Southeast University to evaluate the effectiveness of experimental stimuli. In the present study, the amount of “yes” responses to the protagonist's resolutions for moral dilemmas reflects the participant's preference to utilitarianism. In addition, it has been suggested that reaction time may reflect the deliberation time for one issue (Greene et al., 2004).

Therefore, for the experimental moral dilemma conditions, repeated-measures analysis of variance (ANOVA) was conducted separately for participants' percentage and decision time of “yes” responses to the resolution for moral dilemmas, with resolution type (doing harm, allowing harm, and no harm) as a within-participant factor. For the percentage of “yes” responses to the resolution for moral dilemmas, the main effect of resolution type was significant, *F*_(2,86)_ = 362.94, *p* < 0.001, with the lowest percentage of “yes” responses to protagonist's doing harm behavior (13.14%), followed by allowing harm behavior (27.20%), and then no harm behavior (92.69%), and the differences between any two conditions reached significance, *p* < 0.05. For the decision time of “yes” responses to the resolution for moral dilemmas, the main effect of resolution type was significant, *F*_(2,86)_ = 19.92, *p* < 0.05, with longest RT for doing harm (6,639 ms), followed by RT for allowing harm (4,414 ms), and then RT for no harm (2,928 ms), and the differences between any two conditions reached significance, *p* < 0.05. Both the percentages and decision time of “yes” responses to the resolution for moral dilemmas indicated that, relative to the allowing harm condition, during the doing harm condition, people preferred deontological principles more, suggesting that the action factor affected a participant's behavior response to moral judgment.

In addition, since nearly all the participants made “yes” responses to the resolution under the no harm condition, repeated-measures ANOVA was conducted separately for the decision time of responses to the resolution for moral dilemmas, with response type (yes vs. no) as a within-participant factor only for the doing harm and allowing harm conditions. For the doing harm condition, the main effect of response type was significant, *F*_(1,43)_ = 25.94, *p* < 0.05, with longer RT of “yes” responses (6,639 ms) than RT of “no” responses (2,915 ms). For allowing harm, the main effect of response type also reached significance, *F*_(1,43)_ = 12.82, *p* < 0.05, with longer RT of “yes” responses (4,414 ms) than RT of “no” responses (3,297 ms).

These results supported the deontological principle which claims that factors other than consequences matter, so it is sometimes morally wrong to do what has the best consequences overall (Schaich Borg et al., 2006). Taken together, this standardized set of moral dilemmas based on the DDA is really an effective and normative moral dilemma paradigm, which could be used for future experimental studies on moral judgment.

### Apparatus and Procedure

Before the formal experiment, participants were informed they were going to attend an ERP experiment of context imagination and were required to imagine the story as truly as possible, and then make a judgment under the instruction. EEGs were recorded during the overall procedure of participants reading and judging dilemmas. The participant was seated about 57 cm in front of a 19-inch LCD monitor (screen resolution: 1,024 × 800, refresh rate: 120 Hz, color quality: highest 32 bit) in a dimly lit sound-attenuated room undertaking the EEG test. Each trial began with the presentation of a fixation sign (a small white plus sign subtended 0.7° × 0.7° of visual angle) in the center against a black background. After 800 ms, a paragraph of a scenario was presented, which participants could read at their own pace. When the participants pressed the spacebar, another fixation appeared in the center of the screen. After 500 ms, the “key verb” indicating the protagonist's action was presented for 1,500 ms, followed by the object indicating the object of the protagonist's action. The participants were asked to press button “F” or “J” to select whether they agreed or disagreed with the protagonist's resolution. The agree/disagree buttons were counterbalanced across participants. Then, a blank screen was presented for 1,000 ms.

**Figure 1:**
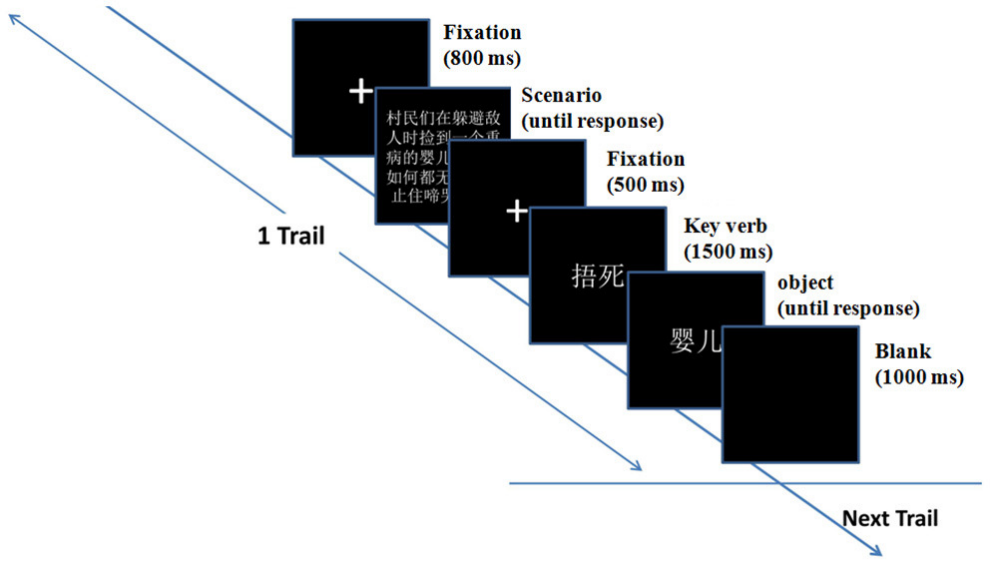
The sequence of events in a single trial.

The ERP experiment task was administered on an Intel Core i3 computer with E-prime 2.0 to control the presentation and timing of stimuli. The formal experiment consisted of six blocks of 32 trials each (30 moral, two filler). For the experimental moral trials, three different types of resolution for one scenario were presented successively but in random order. Participants were allowed to take a short break at the end of each block. A practice block containing six trials was administered before the formal test. The sequence of events in a single trial was shown in Figure 1. The duration of the entire experimental session was about 45 min.

To assess individual variation in moral competency, participants were asked to fill out the moral competence test (MCT, formerly known as the moral judgment test, MJT) (Lind, 2013) after the ERP session. The MCT includes two moral dilemmas: a worker's dilemma and a doctor's dilemma. After presentation of the short story, the participant is first required to judge whether the protagonist's solution was right or wrong on a seven-point Likert scale from −3 to 3, and then asked to rate six arguments supporting (pro-arguments) and six arguments rejecting (counter-arguments) the protagonist's solution in terms of its acceptability on a nine-point Likert scale from “I strongly reject” (−4) to “I strongly accept” (+4). According to the six Kohlbergian stages, each argument represents a certain moral orientation (Colby and Kohlberg, 1987).

The moral competency score (C-score) is calculated as the percentage of an individual's total response variation concerning the moral quality of the given arguments; the calculation method of C-score is described in the literature (Lind, 2008). The C-score reflects the degree to which a participant's judgments about the pro and con arguments are consistent and independent of his/her previously stated opinion (Lind, 1999). The C-score can range from 0 to 100. A C-score of 1–9 is considered very low, 10–19 as low, 20–29 as medium, 30–39 as high, 40–49 as very high, and above 50 is considered extraordinarily high (Lind, 1999, 2008).

### EEG Recording and Analysis

EEGs were recorded from 64 scalp sites using tin electrodes mounted in an elastic cap (NeuroScan Inc., Herndon, VA, USA) according to the international 10–20 system, with the reference on the left mastoid. Eye blinks were monitored with electrodes located above and below the right eye. The horizontal electro-oculogram (EOG) was recorded from electrodes placed 1.5 cm lateral to the left and right external canthi. All electrode impedances were maintained below 10 kΩ. The EEG and EOG were amplified with a band pass of 0.05–70 Hz and continuously sampled at 500 Hz for offline analysis.

Separate EEG epochs of 1,200 ms (with 200 ms pre-stimulus baseline) were extracted off-line and time-locked to the onset of the “key verb” stimuli. Epochs were re-referenced to the linked mastoid electrodes. Ocular artifacts were corrected with an eye movement correction algorithm (Semlitsch et al., 1986). Epochs were baseline-corrected by subtracting from each sample the average activity of that channel during the baseline period. All trials in which EEG voltages exceeded a threshold of ±70 μV during recording were excluded from further analysis. The EEG data were low-pass filtered below 30 Hz.

**Figure 2:**
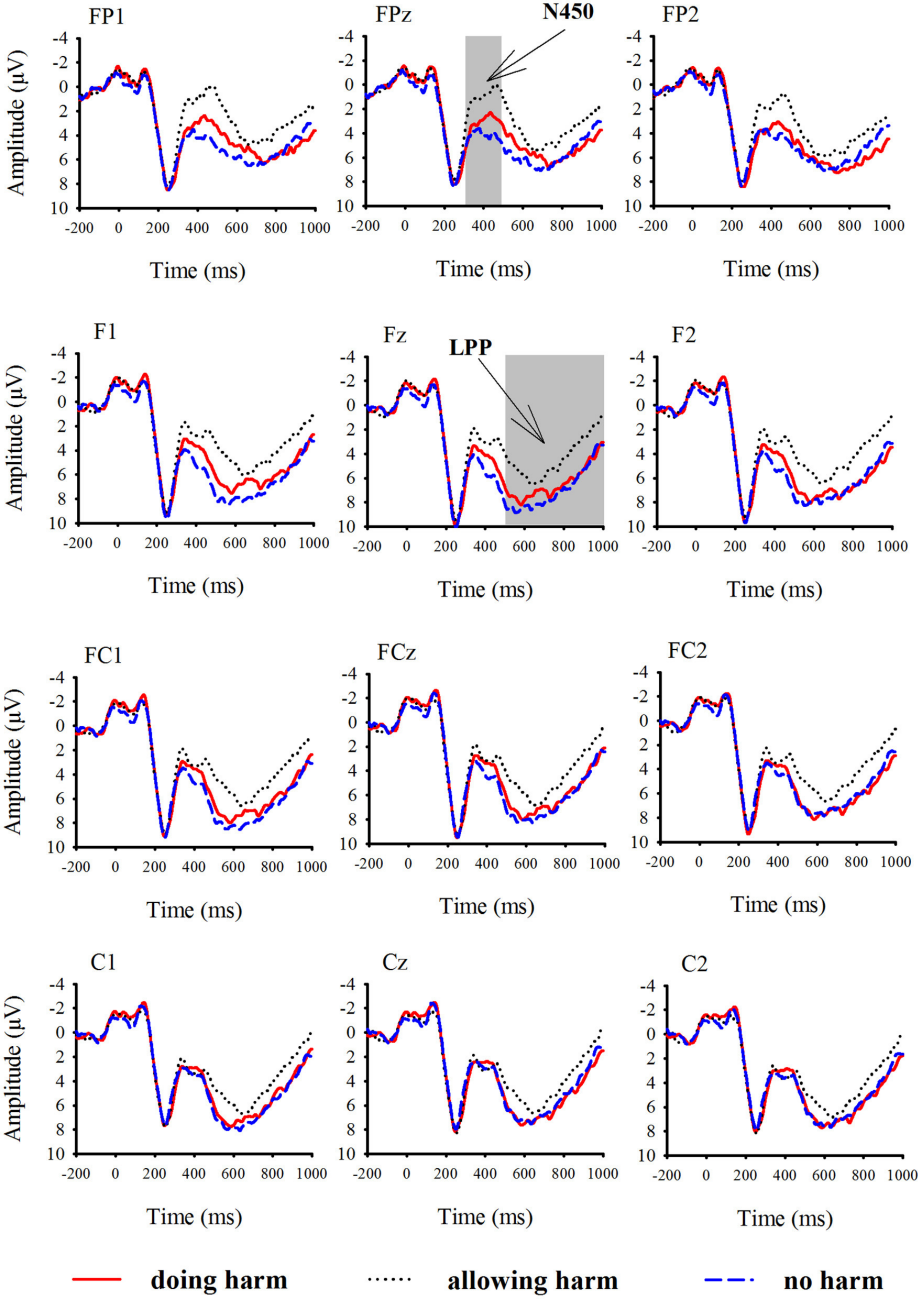
Grand-averaged event-related potential waveforms for doing harm (red lines), allowing harm (black dot lines), and no harm (blue dash lines) conditions at 12 electrodes.

According to visual inspection of grand-averaged waveforms and scalp topographies, the mean amplitudes of the N450 and LPP were measured in the time windows of 300–500 ms and 500–1,000 ms, respectively. For the purpose of statistical analysis, we focused on six prefrontal and frontal electrodes, FP1, FPz, FP2, F3, Fz, and F4, where the amplitudes of N450 were the greatest; we also focused on nine frontal, frontal-central and central electrodes, F3, Fz, F4, FC3, FCz, FC4, C3, Cz, and C4, where the amplitudes of LPP were the greatest. Grand average ERP waveforms are shown in Figure 2.

In this study, the ERPs data were cast into a repeated-measures analysis of variance with three factors for each component: resolution type (doing harm, allowing harm, and no harm) and two electrode position factors (laterality and row). The Greenhouse-Geisser correction for violation of the ANOVA assumption of sphericity was applied where appropriate. The Bonferroni correction was used for multiple comparisons.

sLORETA (standardized low resolution brain electromagnetic tomography) (Pascual-Marqui, 2002) was used to compute the cortical three-dimensional distribution of the potential sources of ERP components that differed between resolution types. The sLORETA is a reliable method that computes images of electric activity from EEG in a realistic head model (Fuchs et al., 2002) using the MNI 152 template (Mazziotta et al., 2001) and estimates three dimensional distribution of current density in 6,239 voxels with a spatial resolution of 5 mm. In the present study, the averaged ERP data within the N450 time frame and the LPP time frame, where the difference between resolution types reached maximum according to the grand-averaged waveforms and scalp distribution, were subjected to sLORETA. The paired *t*-tests on log-transformed data were carried out using sLORETA built-in randomization procedures (5,000 permutations) to correct for multiple comparisons (Nichols and Holmes, 2002). Voxels with *t*-values above the critical threshold (*p* < 0.05) were considered to represent regions of differential activation.

## Results

### Behavior Results

Repeated-measures ANOVA were conducted for the percentage and the decision time of “yes” responses to the resolution for moral dilemmas, with resolution type (doing harm, allowing harm, and no harm) as a within-participant factor. As expected, for the percentage of “yes” responses, the main effect of resolution type was significant, *F*_(2,32)_ = 68.73, *p* < 0.01, with the lowest percentage of “yes” responses to protagonist's doing harm behavior (23.63%), followed by allowing harm behavior (46.86%), and then no harm behavior (93.92%), and the differences between any two conditions reached significance, *p* < 0.05. For the decision time of “yes” responses, the main effect of resolution type was significant, *F*_(2,32)_ = 7.74, *p* < 0.05, with longest RT for doing harm condition (3,302 ms), followed by RT for allowing harm condition (2,518 ms), and then RT for no harm condition (1,276 ms), and the differences between any two conditions reached significance, *p* < 0.05.

Then, due to all the participants being in agreement with the protagonist's resolution under the no harm condition, repeated-measures ANOVA was conducted separately for the decision time of responses to the resolution for moral dilemmas, only for doing harm and allowing harm conditions, with response type (yes vs. no) as a within-participant factor. Specifically, for the doing harm condition, the main effect of response type was significant, *F*_(1,16)_ = 10.06, *p* < 0.05, with longer RT of “yes” responses (3,302 ms) than RT of “no” responses (1,849 ms). However, for the allowing harm condition, the main effect of response type did not reach significance, *p* > 0.05.

### ERP Results

#### The N450

The three-way repeated-measures ANOVA on the mean amplitudes of the N450 over resolution type (doing harm, allowing harm, and no harm), row (prefrontal vs. frontal), and laterality (left, central, and right; see the Method section) revealed a significant main effect of resolution type, *F*_(2,32)_ = 14.01, *p* < 0.05, with ERP responses being more negative for the allowing harm condition (−2.15 μV) than for the doing harm condition (−0.70 μV), followed by the no harm condition (0.67 μV), and the differences between any two conditions reached significance, *p* < 0.05. The main effect of row also reached significance, *F*_(1,16)_ = 5.02, *p* < 0.05, with ERP responses being more negative for the prefrontal areas than for the frontal areas. Neither the other main effects nor interactions reached significance, *p* > 0.1.

#### The LPP

The three-way repeated-measures ANOVA on the mean amplitudes of the LPP over resolution type (doing harm, allowing harm, and no harm), row (frontal, frontocentral, and central), and laterality (left, central, and right; see the Method section) revealed a significant main effect of resolution type, *F*_(2,32)_ = 6.94, *p* < 0.05, with ERP responses being more positive for no harm (6.09 μV) and doing harm conditions (5.97 μV), than for the allowing harm condition (4.34 μV), but the differences between no harm and doing harm conditions did not reach significance, *p* > 0.1. The resolution type also interacted with row and laterality significantly, *F*_(4,64)_ = 6.96, *p* < 0.05, *F*_(4,64)_ = 3.55, *p* < 0.05. The further simple effect analysis showed that, for both the frontal and frontocentral rows rather than the central row, the main effect of resolution type was significant, *p* < 0.05. Specifically, for the frontal row, ERP responses were obviously more positive for no harm (6.64 μV) and doing harm (6.20 μV) conditions than for allowing harm condition (4.26 μV); for the frontocentral row, ERP responses were obviously more positive for the no harm condition (6.19 μV) than for the allowing harm condition (4.67 μV). Therefore, it was evident that the discrepancy among the resolution types only appeared at the anterior area of the brain. In addition, the further simple effect analysis also showed that for all the three lateral lines (i.e., left, middle, and right), the main effect of resolution type was significant, *p* < 0.05. Specifically, for the left line, ERP responses were evidently more positive for the no harm condition (6.31 μV) than for the allowing harm condition (4.31 μV); for the midline, ERP responses were evidently more positive for the no harm (6.06 μV) and doing harm (5.90 μV) conditions than for the allowing harm condition (4.27 μV). For the right line, ERP responses were more positive for the no harm condition (5.90 μV) than for the allowing harm condition (4.44 μV). Neither of the other main effects nor interactions reached significance, *p* > 0.1.

### Current Source Density Analysis Using sLORETA

It is clear that in both the N450 and LPP mean amplitude analysis, the significant main effects of resolution type were found. Thus, the sLORETA-images were compared (a) between allowing harm and doing harm conditions; (b) between allowing harm and no harm conditions; and (c) between doing harm and no harm conditions in the time frame of 440–450 ms, and 590–600 ms, respectively, where the difference among resolution types reached maximum according to the grand-averaged waveform.

**Figure 3:**
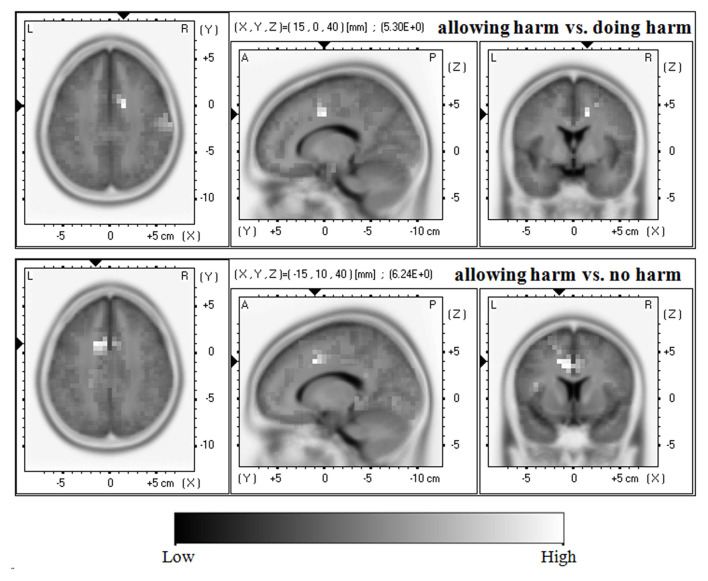
Horizontal, sagittal, and coronal slices of sLORETA maps for the N450 at 440–450 ms for the contrast: between allowing harm and doing harm (upper map, max: cingulate gyrus, middle frontal gyrus), between allowing harm and no harm (lower map, max: cingulate gyrus, middle frontal gyrus, medial frontal gyrus).

As shown in Figure 3, in terms of the N450, compared with the doing harm condition, the allowing harm condition showed greater current density in the cingulate gyrus (BA24/32/23) and the middle frontal gyrus (BA6/8). In addition, compared with the no harm condition, the allowing harm condition showed higher current density in the cingulate gyrus (BA32/24/6), the middle frontal gyrus (BA8), and the medial frontal gyrus (BA9). However, the current source density difference between doing harm and no harm conditions did not reach significance.

**Figure 4:**
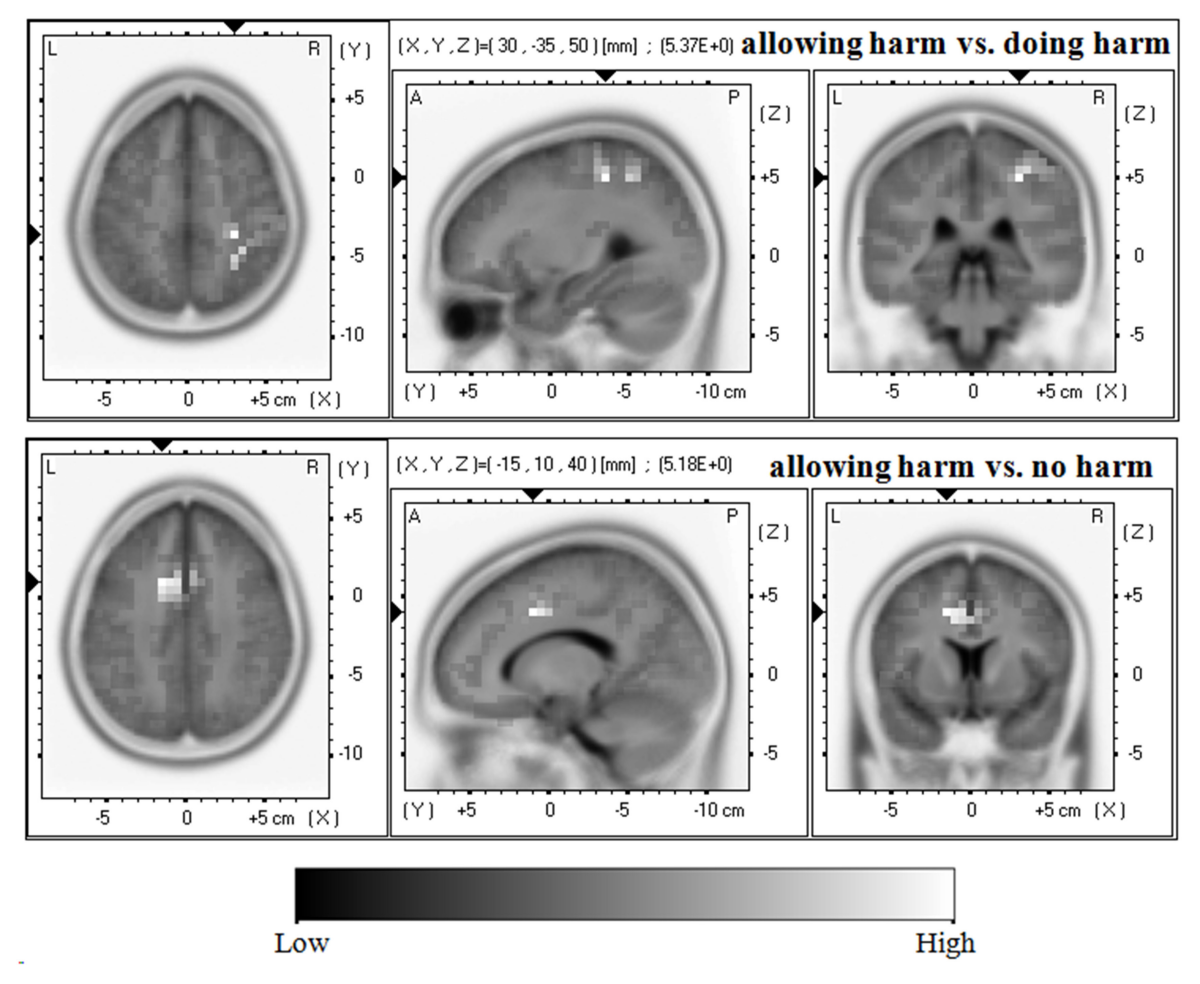
Horizontal, sagittal, and coronal slices of sLORETA maps for the LPP at 590–600 ms for the contrast: between doing harm and allowing harm (upper map, max: postcentral gyrus, inferior parietal lobule, cingulate gyrus), between no harm and allowing harm (lower map, max: cingulate gyrus, middle frontal gyrus, medial frontal gyrus).

As shown in Figure 4, with regard to the LPP, compared with the allowing harm condition, the doing harm condition showed greater current density in the postcentral gyrus (BA3/2), the precentral gyrus (BA4), the inferior parietal lobule (BA40), and the cingulate gyrus (BA31). In addition, compared with the allowing harm condition, the no harm condition showed higher current density in the cingulate gyrus (BA32/24/6), the middle frontal gyrus (BA8), and the medial frontal gyrus (BA9). However, the current source density difference between doing harm and no harm conditions did not reach significance.

### Correlations of Behavior and ERP Data

The mean C-score for all participants was 17.38 (SD = 10.57). The LPP amplitude difference between doing harm and allowing harm was positively correlated with C-score, *R* = 0.80, *p* < 0.001, and the LPP amplitude difference between no harm and allowing harm conditions was also positively correlated with C-score, *R* = 0.60, *p* < 0.05.

## Discussion

Utilitarianism (also called consequentialism) and deontological principles are two important and opposite moral theories. Two ERP studies have successfully explored the temporal dynamics of emotional and cognitive processing during decision-making in a set of classic moral dilemmas inspired by the DDE, which follows deontological principles, also known as the intention principle (Sarlo et al., 2012; Pletti et al., 2015). The results suggested that the intention factor can modulate both the affective and cognitive processing of moral judgment (Sarlo et al., 2012). Here, we newly developed a moral dilemma paradigm on the basis of the DDA, which is another type of deontological principles (i.e., harm caused by action is morally worse and more unacceptable than harm caused by omission) to investigate the time course of neural responses to moral judgment and to examine how the action factor modulates the temporal dynamics of moral judgment. Our paradigm can separate reading and encoding processes from decision-making and thus be appropriate for ERP experiment. Behavior results showed that participants made significantly higher percentages of utilitarian judgments for the allowing harm condition than for the doing harm condition. Moreover, when approving of the protagonist's utilitarian options, participants spent more reaction time on the doing harm resolution than on the allowing harm resolution. In addition, our reaction time data even exhibited that participants spent more time on making utilitarian judgments than on making deontological judgments only for the doing harm condition. ERP and sLORETA results revealed that the N450 amplitude reached its most negative value for the allowing harm condition, followed by the doing harm condition, and then the no harm condition. The allowing harm condition evoked a larger current density in the cingulate gyrus, middle frontal gyrus, and medial frontal gyrus than both the doing harm and no harm conditions. The LPP amplitude was significantly more positive for the doing harm and no harm conditions than for the allowing harm condition. The doing harm condition evoked a larger current density in the precentral gyrus, inferior parietal lobule, and cingulate gyrus than the allowing harm condition, and the no harm condition evoked a larger current density in the cingulate gyrus, middle frontal gyrus, and medial frontal gyrus than the allowing harm condition. In the following paragraph, we discuss the implications of these findings in terms of moral judgment guided by the DDA.

The moral dilemma task developed by Greene et al. (2001) and its revised task are frequently used to investigate psychological and neural responses to utilitarian and deontological judgments on moral dilemmas. These moral dilemma tasks can be further refined into two dilemma types, i.e., “personal” (or “Footbridge-like”) and “impersonal” (or “Trolley-like”) dilemma. However, in recent years, more and more researchers have questioned this set of dilemmas. Firstly, just as Greene's initial emphasis on such coarse personal/impersonal distinction (Greene et al., 2001), this distinction has not been regarded as a philosophically accurate formulation of the deontological distinction between the Trolley and Footbridge scenarios (Kahane and Shackel, 2010), and is likely derived from the DDA or something similar (Cushman et al., 2006; Schaich Borg et al., 2006). Accordingly, it is obvious that personal/impersonal dilemmas lack an appropriate moral theories basis. Secondly, Schaich Borg et al. (2006) highlighted that the language used to describe the moral personal scenarios was more emotive or colorful than that used to describe the non-moral or moral impersonal scenarios in this set of dilemmas (Schaich Borg et al., 2006; Kahane and Shackel, 2010). Last but not least, the way of asking questions in this set of dilemmas, like “the participants were asked whether a certain act is appropriate”, may produce the risk that different individuals would have different questions in mind (Schaich Borg et al., 2006; Mikhail, 2007; Kahane and Shackel, 2010). To overcome the abovementioned deficits, we tried to develop a novel set of moral dilemmas on the basis of a classical deontological principle, namely, the DDA, employing non-colorful language and phrasing questions in appropriate normative vocabulary.

Behaviorally, a lower percentage and longer RT of “yes” responses for the doing harm condition than for the allowing harm condition supported the DDA powerfully, referring to harm caused by omission being more acceptable than equal harm caused by acts (Baron and Ritov, 2004). Additionally, only for the doing harm condition, participants spent more time on “yes” responses (i.e., agreeing with the utilitarian judgment) than “no” responses (i.e., agreeing with the deontological judgment). Such an RT differentiation pattern resembled the pattern which is reported in several studies on moral utilitarianism and deontology. Greene et al. (2001) first presented that the participants responded more slowly to utilitarian judgments than to deontological judgments when dealing with the personal dilemmas. The authors explained that people tend to have a salient automatic emotional response to the personal dilemmas, leading them to judge the action to be inappropriate. A few people who nevertheless judge this action to be appropriate rationally may override such an emotional interference, therefore exhibiting a longer reaction time to judgment (Greene et al., 2001). Tremoliere and Bonnefon (2014) admitted that utilitarian responses demand the deliberate thinking system to override intuitively cued emotional deontological responses, furthermore they even claimed that efficient kill-save ratios can promote utilitarian responding and ease up the cognitive demands. Analogously, such emotional interference in the personal condition may exist in the doing harm condition of the present experiment. Doing harm may elicit a stronger emotional response and most people are inclined to reject such a resolution intuitively according to the deontological principle. The few participants who agree with the doing harm resolution for consequentialism have to recruit cognitive control to overcome emotional interference, leading to longer reaction times to judgment for the doing harm condition than for the allowing harm condition. Therefore, our behavior data concerning participant's selection and reaction time may strongly support the dual-processing theory in that moral judgments are the product of two competing processing systems; one is a fast and automatic emotional system driving deontological judgments, and the other is a slow and controlled cognitive system favoring utilitarian judgments (Greene et al., 2001, 2004, 2008; Martínez et al., 2020).

In terms of the ERP results, the N450 amplitude reached its most negative value in the allowing harm condition, followed by the doing harm condition, then the no harm condition. Such differences were localized at the cingulate gyrus (BA24/32/23), middle frontal gyrus (BA6/8), and medial frontal gyrus (BA9). These findings are in accordance with the perspective that cognitive control is involved in moral judgment on moral dilemmas. It has been consistently found that the N450, which reflects the cognitive control processes and/or cognitive effort, can be modulated by changes in cognitive control (Stock et al., 2016; Zink et al., 2018). In addition, many studies have demonstrated that increased activities in the DLPFC and ACC reflect the cognitive control processes and/or cognitive effort during cognitive and affective conflict (Ochsner et al., 2009), even social and economic conflict (Baumgartner et al., 2011; Koban et al., 2014; Feng et al., 2015). Importantly, the role of the brain areas related to cognitive control, i.e., DLPFC and ACC, has been emphasized in utilitarian moral judgment on moral dilemmas (Greene et al., 2001, 2004; Koop, 2013). Therefore, it can be easily inferred that a great N450 amplitude will be elicited when making utilitarian moral dilemma judgments. The findings that more participants made utilitarian selections and their N450 amplitudes were more negative source localized at cognitive control brain areas when faced with the harm caused by omission (i.e., allowing harm) situation than when facing with the harm caused by action (i.e., doing harm) situation fits such an inference well.

For the later component LPP, the protagonists' doing harm and no harm behavior elicited a more positive amplitude than their allowing harm behavior. Moreover, relative to the allowing harm condition, the activities of the LPP in the doing harm condition were mainly localized in the primary motor cortex (BA4) and postcentral gyrus (BA3/2) related to motor imagery and motor learning (Hétu et al., 2013), the supramarginal gyrus (BA40) associated with complex semantic processing (Stoeckel et al., 2009) and working memory (Deschamps et al., 2014), and the dorsal posterior cingulate (BA31) involved in the dorsal attention network and related to top-down control of attention (Leech et al., 2011). Previous ERP studies on moral judgment have suggested the parietal LPP should be assumed to be proportional to the attentional distribution and working memory load, due to the greater cortical positivity during decisions on incidental than instrumental dilemmas at more posterior locations, particularly in the time window of 450–600 ms (Sarlo et al., 2012; Pletti et al., 2015). Therefore, the differentiation of LPP amplitude and its spatial activities between doing harm and allowing harm conditions may reflect the high cognitive processes, like distribution of attention resources, semantic processing, and working memory. In addition, compared with the allowing harm condition, the activities of the LPP in the no harm condition were mainly localized in the cingulate gyrus (BA32/24/6) which is involved in behavioral inhibition (Coderre et al., 2008), frontal eye fields (BA8) partially involved in executive functions (Kübler et al., 2006), and dorsolateral prefrontal cortex (BA9) related to cognitive control (MacDonald et al., 2000). Specifically, previous fMRI studies have suggested that the right dorsolateral prefrontal cortex (rdlPFC) plays an important role in cognitive control processes required for a cost–benefit computation that eventually leads to utilitarian judgments (Greene et al., 2001, 2004; Schaich Borg et al., 2006; Wei et al., 2010). Here, in our experiment, the behavior results have showed that most participants selected the utilitarian judgment for the no harm condition rather than for the allowing harm condition. The difference of the LPP amplitudes and its activations in brain regions between no harm and allowing harm conditions may reflect the cognitive control processing in moral judgment. Furthermore, considering the further correlation between the LPP amplitude and the C-score of MCT which indexed the cognitive aspect of moral judgment competence, we suggested that the LPP might reflect attentional allocation and cognitive “rational” control processes during moral judgment.

What's more, previous results suggested that the temporal dynamics of cognitive-emotional interplay in moral judgments can be mainly divided into two stages, i.e., one is the early stage indexed by the frontal P260, reflecting an immediate affective appraisal of the available options; the other is the later stage indexed by parietal complex slow waves, reflecting attentional resource distribution and working memory load (Sarlo et al., 2012; Pletti et al., 2015). In our study, we merely found the late ERP components associated with cognitive processing, i.e., the N450 and the LPP were modulated by the resolution type in different degrees, whereas the frontal P260 was not modulated by this factor. Therefore, replicating the viewpoint suggested by Cushman et al. (2006), these results may indicate that action factor only modulates the conscious reasoning processing rather than the automatic processing of moral judgments.

## Conclusion

In short, the present study investigated the behavior and spatial temporal cortical activation of moral judgments with a newly-developed moral dilemma paradigm under the framework of the DDA. Behavior data showed that participants preferred utilitarian judgments and spent less time for the allowing harm condition than for the doing harm condition. In addition, only for the doing harm condition, participants responded more slowly to the utilitarian judgment than to the deontological judgment. ERP and sLORETA results mainly revealed that, compared to the doing harm and no harm dilemmas, the allowing harm dilemmas elicited the greatest N450 which evoked a larger current density at brain areas associated with cognitive control. The LPP amplitude was significantly more positive for both the doing harm and no harm conditions than for the allowing harm condition, and these differences were positively correlated with C-score which indexed the cognitive aspect of moral judgment competence. In addition, the related activation differences were mainly at brain regions associated with top-down control of attention and cognitive “rational” control processes. These findings suggested that people have a strong omission bias during moral judgment. Furthermore, the action factor modulates the conscious reasoning processing of moral judgment, including the conflict detection, attentional allocation, and cognitive “rational” control processes during moral judgment.

## Data Availability Statement

The raw data supporting the conclusions of this article will be made available by the authors, without undue reservation.

## Ethics Statement

The studies involving human participants were reviewed and approved by the Ethics Committee of Affiliated Zhongda Hospital, Southeast University, China. The patients/participants provided their written informed consent to participate in this study.

## Author Contributions

YL, JZ, YZ, and XY: conception and design. JZ, YZ, and XY: collection of data. JZ: analysis and interpretation of data. YL: drafting and revising the article. All authors: contributed to the article and approved the submitted version.

## Conflict of Interest

The authors declare that the research was conducted in the absence of any commercial or financial relationships that could be construed as a potential conflict of interest.
