# Supplementary material for: Action Modulates the Conscious Reasoning Process of Moral Judgment: Evidence From Behavior and Neurophysiology
Source: Front Behav Neurosci. 2021 Jan 6;14:577252. doi: 10.3389/fnbeh.2020.577252 (PMC7815760; doi:10.3389/fnbeh.2020.577252)
Supplement: Supplementary file 1 [file Data_Sheet_1.PDF]

**Experimental moral dilemmas in English**				
**No.**	**Resolution type**	**Scenario**	**Protagonist's action**	**Object of the protagonist's action**
1	Doing harm	An emergency physician additionally receives five slightly injured patients when giving a blood transfusion to a critically injured patient. If the patients with slight injury do not receive blood transfusion in time, their conditions will deteriorate and they will die, but the blood bank almost runs out of blood at this time. In order to save more lives, the doctors take some actions on the patient with serious injury. Five patients with slight injury are saved, but the patient with serious injury dies. The actions taken by the doctor are……	Cutting off	Blood transfusion
	Allowing harm	An emergency physician additionally receives five slightly injured patients when giving a blood transfusion to a critically injured patient. If the patients with slight injury are not treated in time in time, their conditions will deteriorate and they will die, but the manpower is in shortage at this time. In order to save more lives, the doctors take some actions on the patient with serious injury. Five patients with slight injury are saved, but the patient with serious injury dies. The actions taken by the doctor are……	Giving up	Treatment
	No harm	An emergency physician additionally receives five slightly injured patients when giving a blood transfusion to a critically injured patients. If the patients with slight injury are not treated in time, their conditions will deteriorate and they will die, and colleagues of the doctor are available at this time. In order to save more lives, the doctors take some actions on the patient with serious injury. Five patients with slight injury are saved, but the patient with serious injury dies. The actions taken by the doctor are……	Asking for	Assistance

2	Doing harm	A biologist triggers an extremely dangerous virus in an experiment. Not promptly closing the security gate would lead to the spread of the virus and threaten more people's lives. At that moment, the biologist has not successfully escaped. In order to close the security gate in time, students of the biologist take some actions. Eventually, the virus is under control but the biologist dies. The actions taken by the student are ...	Closing	The door
	Allowing harm	A biologist triggers an extremely dangerous virus in an experiment. Opening the door to rescue the biologist would lead to the spread of the virus and threaten more people's lives. At that moment, the biologist has fallen in a faint. In order to close the security gate in time, students of the biologist take some actions. Eventually, the virus is under control but the biologist dies. The actions taken by the student are ...	Giving up	Opening the door
	No harm	A biologist triggers an extremely dangerous virus in an experiment. Not promptly closing the security gate would lead to the spread of the virus and threaten more people's lives. At that moment, the biologist has teetered. In order to close the security gate in time, students of the biologist takes some actions. Eventually, the virus is under control but the biologist dies. The actions taken by the student are ...	Rescuing	The teacher
3	Doing harm	The eldest son with mental illness named "A Niu" holding his younger brother jumps into the river. His father cannot rescue the younger brother because “A Niu” hold him tightly,. In order not to lose the younger healthy son, the father takes some actions. Eventually, the youngest son is saved but “A Niu” dies. The actions taken by the father are ...	Drowning	A Niu
	Allowing harm	The eldest son with mental illness named "A Niu" holding his younger brother jumps into the river. Because of the turbulent river, his father cannot rescue both of them at the same time. In order not to lose the younger healthy son, the father	Giving up	A Niu

		takes some actions. Eventually, the younger son is saved but “A Niu” dies. The actions taken by the father are ...		
	No harm	The eldest son with mental illness named "A Niu" holding his younger brother jumps into the river. As the father is not far from them, he noticed the situation in time. In order not to lose his children, the father takes some measures. Eventually, the younger son is saved but “A Niu” dies. The actions taken by the father are ...	Rescuing	Both
4	Doing harm	An old man with dementia and his grandson are trapped by a fire. The old man holds the child tightly and refuses to escape. In order to save the child's life, the old man's son takes some actions. Eventually, the child is saved but the old man dies. The actions taken by the old man’s son are ...	Knocking out	His father
	Allowing harm	An old man with dementia and his grandson are trapped by a fire. The old man’s son cannot save both of them at the same time. In order to save the child's life, the old man's son takes some actions. Eventually, the child is saved but the old man dies. The actions taken by the old man’s son are ...	Giving up	His father
	No harm	An old man with dementia and his grandson are trapped by a fire . The old man’s son immediately arrives after hearing of this situation. In order to save their lives, the old man's son takes some actions. Eventually, the child is saved but the old man dies. The actions taken by the old man’s son are ...	Carrying	Both
5	Doing harm	The villagers find a seriously ill baby when dodging the enemies, but in any case they cannot stop her from crying. If the baby's cry does not stop in time, all villagers’ lives will be threatened. In order to prevent the crying attracting the enemies, the village chief takes some actions. Eventually, the villagers safely hide from the enemies, but the baby dies. The actions taken by the village chief are ...	Smothering	The baby
	Allowing harm	The villagers find a seriously ill baby when dodging the enemies, but in any case they cannot stop her from crying. If carrying the crying baby, all villagers’ lives will be threatened. In order to prevent the crying attracting the enemies, the	Giving up	The baby 33

		village chief takes some actions. Eventually, the villagers safely hide from the enemies, but the baby dies. The actions taken by the village chief are ...		
	No harm	The villagers find a seriously ill baby when dodging the enemies, and the village chief is the only one who can stop her crying. If the baby does not stop crying in time, all villagers' lives will be threatened. In order to prevent the crying attracting the enemies, the village chief takes some actions. Eventually, the villagers safely hide from the enemies, but the baby dies. The actions taken by the village chief are ...	Soothing	The baby
6	Doing harm	When dodging the enemies, a new recruit falls into the hunters' trap by mistake, and is seriously injured. At this moment, the enemies are approaching. If no one rolls down the hillside to distract the enemies, everyone will be in danger. In order to save more soldiers' lives, the captain of the recruits takes some actions. Eventually, the other soldiers dodge the enemies safely, but that recruit dies. The actions taken by the captain of the recruits are... ...	Pushing	The recruit
	Allowing harm	When dodging the enemies, a new recruit falls into the hunter's trap by mistake, and is seriously injured. At this moment, the enemies are approaching. If fleeing with this injured recruit, everyone will be in danger. In order to save more soldiers' lives, the captain of the recruits takes some actions. Eventually, the other soldiers are safe, but that recruit dies. The actions taken by the captain of the recruits are...	Giving up	The recruit
	No harm	When dodged the enemies, a recruit falls into the hunters' trap by mistake, and is seriously injured. At this moment, the enemies are approaching. There is not enough time to carry this recruit to flee, and the time only allows him to hide. In order to save more soldiers' lives, the captain of the recruits takes some actions. Eventually, the other soldiers are safe, but that recruit dies. The actions taken by the captain of the recruits are...	Hiding	The recruit

7	Doing harm	A fishing boat gets lost at sea for many days, and has been short of food. One of the crews has been as weak as a cat. If the other crews continue to wait for rescue, they can only eat that weak crew's flesh to sustain their lives. In order to save more lives, the captain takes some actions on that weak crew. Eventually, the other crews are saved but that weak crew dies. The actions taken by the captain against the crew are ...	Eating	The crew
	Allowing harm	A fishing boat gets lost at sea for many days, and has been short of food. One of the crews has been as weak as a cat. If the other crews conserve energy to wait for rescue, they cannot take care of that weak crew. In order to save more lives, the captain takes some actions on that weak crew. Eventually, the other crews are saved but that weak crew dies. The actions taken by the captain against the crew are ...	Ignoring	The crew
	No harm	A fishing boat gets lost at sea for many days, and has been short of food. One of the crews has been as weak as a cat. If the other crews continue to wait for rescue, their belief in survival should not be shaken. In order to save more lives, the captain takes some actions to that weak crew. Eventually, the other crews are saved but that weak crew dies. The actions taken by the captain against the crew are ...	Encouraging	The crew
8	Doing harm	A captain and several tourists have been adrift to a deserted island since their ship was sunk. They are lack of food. One of the tourists is seriously injured. If they want to survive, they can only eat partners' flesh to sustain their lives. In order to save more lives, the captain takes some actions on the injured tourist. Eventually, the others are saved but that injured tourist dies. The measures taken by the captain on the tourist are ...	Eating	The tourist
	Allowing harm	A captain and several tourists have been adrift to a deserted island since their ship was sunk. They are lack of food. One of the tourists is seriously injured. If they	Ignoring	The tourist

		want to survive, they need to conserve physical strength. In order to save more lives, the captain takes some actions to the injured tourist. Eventually, the others are saved but that injured tourist dies. The measures taken by the captain on the tourist are ...		
	No harm	A captain and several tourists have been adrift to a deserted island since their ship was sunk. They are lack of food. , one of the tourists seriously injured. If they want to survive, they need to maintain the belief in survival. In order to save more lives, the captain takes some actions to the injured tourist. Eventually, the others are saved but that injured tourist dies. The actions taken by the captain on the tourist are ...	Encouraging	The tourist
9	Doing harm	A man who has just rescued a little girl from the flood to the board encounters an old man who needs help. The board is too fragile to bear the weight of both of the little girl and the old man. For the little girl's safety, the man takes some actions on the old man’s hand that seizes the board. Eventually, the little girl is saved, but the old man dies. The actions taken by the man are ...	Removing	The fingers
	Allowing harm	A man who has just rescued a little girl from the flood to the board encounters an old man who needs help. The board is too fragile to bear the weight of both of the little girl and the old man. For the little girl's safety, the man takes some actions on the old man who is going to drown. Eventually, the little girl is saved, but the old man dies. The actions taken by the man are ...	Giving up	Old man
	No harm	A man who has just rescued a little girl from the flood to the board encounters an old man who needs help. The board is too fragile, and a driftwood comes by. For the little girl's safety, the man takes some actions on the old man who is going to drown. Eventually, the little girl is saved, but the old man dies. The actions taken by men are ...	Pulling onto	The driftwood
10	Doing harm	A surgery of a seriously sick woman is failed and unbearable pain tortures her. In	Smothering	The woman

		order to alleviate her suffering, her husband takes some actions. Eventually, the woman dies. The actions taken by her husband are ...		
	Allowing harm	A surgery of a seriously sick woman is failed and extra surgery cannot help her either. In order to alleviate her suffering, her husband takes some actions. Eventually, the woman died. The actions taken by her husband are ...	Giving up	The Treatment
	No harm	A surgery of a seriously sick woman is failed and she is desperate. In order to alleviate her suffering, her husband takes some actions. Eventually, the woman dies. The actions taken by her husband are …	Accompanyin g	The woman
11	Doing harm	A soldier falls into the enemy's trap when executing a secret mission. The enemy would certainly torture him to obtain information. For the safety of more people, the captain takes some actions on the soldier. Eventually, the others are out of danger, but that soldier dies. The actions taken by the captain are ...	Killing	The soldier
	Allowing harm	A soldier falls into the enemy's trap when executing a secret mission. There is not enough time to save this soldier. For the safety of more people, the captain takes some actions on the soldier. Eventually, the others are out of danger, but that soldier dies. The actions taken by the captain are ...	Giving up	The soldier
	No harm	A soldier falls into the enemy's trap when executing a secret mission. The captain has some knowledge about how to disarm this trap. For the safety of more people, the captain takes some actions on the soldier. Eventually, the others were out of danger, but that soldier dies. The actions taken by the captain are ...	Rescuing	The soldier
12	Doing harm	At midnight, a toxic gas leaks from the medical laboratory and automatically exhausts into a double room. An old man with cancer is wearing an oxygen mask while a young patient does not. In order to save the life of the young patient, the watchman takes some actions on the old man with cancer. Eventually, the young patient is saved, but the old man with cancer dies. The actions taken by the watchman on the old man with cancer are ...	Taking away	The mask

	Allowing harm	At midnight, a toxic gas leaks from the medical laboratory and automatically exhausts into a double room. It is in emergency, and the watchman cannot save two patients at the same time. In order to save the life of the young patient, the watchman takes some actions on the old man with cancer. Eventually, the young patient is saved, but the old man with cancer dies. The actions taken by the watchman on the old man with cancer are ...	Giving up	The old man
	No harm	At midnight, a toxic gas leaks from the medical laboratory and automatically exhausts into a double room. The watchman cannot save two patients at the same time but his colleague just arrives and is going to take his turn on duty. In order to save the lives of both the old man and the young man, the watchman takes some actions on the colleague. Eventually, the young patient is saved, but the old man with cancer dies. The actions taken by the watchman on the colleague are ...	Asking for	The assistance
13	Doing harm	Police arrest the criminal who has installed time bombs in highly populated areas. At this moment, the criminal's pregnant wife is nearby. In order to force the criminal to confess the place where the bombs are hidden as soon as possible, a policeman takes some actions on the criminal’s wife. Eventually, the police find the bombs in time, but the criminal's wife is dying. The actions taken by the policeman are ...	Beating	The pregnant woman
	Allowing harm	Police arrest the criminal who has installed time bombs in highly populated areas. At this moment, the criminal's wife gives birth prematurely suddenly. In order to force the criminal to confess the place where the bombs are hidden as soon as possible, a policeman takes some actions on the criminal’s wife. Eventually, the police find the bombs in time, but the criminal's wife is dying. The actions taken by the policeman are ...	Ignoring	The pregnant woman
	No harm	Police arrest the criminal who has installed time bombs in highly populated areas. At this moment, the criminal's wife gives birth prematurely suddenly. In order to	Helping	The pregnant woman

		motivate the criminal to confess the place where the bombs are hidden as soon as possible, a policeman takes some actions on the criminal’s wife. Eventually, the police find the bombs in time, but the criminal's wife is dying. The actions taken by the policeman are ...		
14	Doing harm	Police arrest the criminal who has installed time bombs in highly populated areas. At this moment, the criminal's little daughter is nearby. In order to force the criminal to confess the place where the bombs are hidden as soon as possible, a policeman takes some actions on the criminal’s little daughter. Eventually, the police find the bombs in time, but the criminal's daughter is dying. The actions taken by the policeman are ...	Beating	The girl
	Allowing harm	Police arrest the criminal who has installed time bombs in highly populated areas. At this moment, the criminal's little daughter has an asthma attack. In order to force the criminal to confess the place where the bombs are hidden as soon as possible, a policeman takes some actions on the criminal’s little daughter. Eventually, the police find the bombs in time, but the criminal's daughter is dying. The actions taken by the policeman are ...	Ignoring	The girl
	No harm	Police arrest the criminal who has installed time bombs in highly populated areas. At this moment, the criminal's little daughter had an asthma attack. In order to motivate the criminal to confess the place where the bombs are hidden as soon as possible, a policeman takes some actions on the criminal’s little daughter. Eventually, the police find the bombs in time, but the criminal's daughter is dying. The actions taken by the policeman are ...	Helping	The girl
15	Doing harm	After the typhoon, a trunk falls onto the tiger’s enclosure in the zoo. The feeder must remove the trunk as soon as possible to prevent the tiger from climbing out of the enclosure along the trunk and threatening the safety of many tourists. At this moment, a tourist stands next to the tiger’s enclosure. In order to buy time to	Pushing down	The tourist

		remove the trunk, the feeder takes some actions on this tourist. Eventually, the trunk is removed in time, but the tiger kills the tourist. The actions taken by the feeder are ...		
	Allowing harm	After the typhoon, a trunk falls onto the tiger's enclosure in the zoo. The feeder must remove the trunk as soon as possible to prevent the tiger from climbing out of the enclosure along the trunk and threatening the safety of many tourists. At this moment, a tourist falls into the tiger enclosure by accident. In order to be hurry to remove the trunk, the feeder takes some actions on this tourist. Eventually, the trunk is removed in time, but the tiger kills the tourist. The actions taken by the feeder are ...	Ignoring	The tourist
	No harm	After the typhoon, a trunk falls onto the tiger's enclosure in the zoo. The feeder must remove the trunk as soon as possible to prevent the tiger from climbing out of the enclosure along the trunk and threatening the safety of many tourists. At this moment, a tourist persists to stand close to the tiger enclosure. In order to save this tourist's life, the feeder takes some actions on this tourist. Eventually, the trunk is removed in time, but the tiger kills the tourist. The actions taken by the feeder are ...	Warning	The tourist
16	Doing harm	The zoo's decorator accidentally dumps the wood on the lion's enclosure in the zoo. The feeder must remove the wood as soon as possible to prevent the lion from climbing outside of the enclosure along the wood to attack the tourists. At this moment, a tourist stands next to the lion's enclosure. In order to draw away the lion to remove the wood, the feeder takes some actions on this tourist. Eventually, the wood is removed in time, but the lion kills the tourist. The actions taken by the breeder are ...	Pushing down	The tourist
	Allowing harm	The zoo's decorator accidentally dumps the wood on the lion's enclosure of in the zoo. The feeder must remove the wood as soon as possible to prevent the lion	Ignoring	The tourist

		from climbing outside of the enclosure along the wood to attack the tourists. At this moment, a tourist falls into the lion’s enclosure by accident. In order to draw away the lion to remove the wood, the feeder takes some actions on this tourist. Eventually, the wood is removed in time, but the lion kills the tourist. The actions taken by the breeder are ...		
	No harm	The zoo's decorator accidentally dumps the wood on the lion’s enclosure of in the zoo. The feeder must remove the wood as soon as possible to prevent the lion from climbing outside of the enclosure along the wood to attack the tourists. At this moment, a tourist persists to stand close to the lion’s enclosure. In order to save this tourist’s life, the feeder takes some actions on this tourist. Eventually, the wood is removed in time, but the lion kills the tourist. The actions taken by the breeder are ...	Warning	The tourist
17	Doing harm	A captain leads the passengers to the lifeboat after the ship struck a rock. But after the last man who is very fat gets aboard, the lifeboat overloads and begins to sink. In order to save the lives of more passengers, the captain takes some actions on to the last fat man. Eventually, most passengers are saved, but the last fat man is drowned. The actions taken by the captain are ...	Pushing down	The big fat
	Allowing harm	A captain leads the passengers to the lifeboat after the ship struck a rock. When the last man who is very fat is going to board the lifeboat, there is no space on the lifeboat. In order to save the lives of more passengers, the captain takes some actions on last fat man. Eventually, most passengers are saved, but the last fat man is drowned. The actions taken by the captain are ...	Giving up	The big fat
	No harm	A captain leads the passengers to the lifeboat after the ship struck a rock. When a seriously ill fat man is boarding the lifeboat, there is still enough space on the lifeboat. In order to save the lives of more passengers, the captain takes some actions on the fat man. Eventually, most passengers are saved, but the fat man is	Helping	The big fat

		drowned. The actions taken by the captain are ...		
18	Doing harm	The ship's lifeguard finds a shark swimming to five tourists who are playing in the sea. At this moment, a young man on board is accidentally scratched and bleeding. In order to draw away the shark and to save more lives, the lifeguard takes some actions on the bleeding young man. Eventually, five tourists are rescued, but the shark swallows the young man. The actions taken by the lifeguard are ...	Pushing down	The young man
	Allowing harm	The ship's lifeguard finds a shark swimming to five tourists who are playing in the sea. At this moment, a young man on board falls into the sea accidentally. In order to buy time to save more lives, the lifeguard takes some actions on the young man. Eventually, five tourists are rescued, but the shark swallows the young man. The actions taken by the lifeguard are ...	Ignoring	The young man
	No harm	The ship's lifeguard finds a shark swimming to five tourists who are playing in the sea. At this moment, a young man on board is holding the fresh chicken, which may attract the shark. In order to save the five tourists and keep the young man safe, the lifeguard takes some actions on the young man. Eventually, five tourists are rescued, but the shark swallows the young man. The actions taken by the lifeguard are ...	Reminding	The young man
19	Doing harm	The teacher and students survived in the tsunami meet a seriously injured man. The man climbs onto their small boat and asks for help. In order to send the children to a safe place as soon as possible, the teacher takes some actions. Eventually, the children are rescued but the injured man dies. The actions taken by the teacher are...	Pushing down	The man
	Allowing harm	The teacher and students survived in the tsunami meet a man in coma. The teacher has been unable to save others. In order to send the children to a safe place as soon as possible, the teacher takes some actions. Eventually, the children	Giving up	The man

		are rescued but the injured man dies. The actions taken by the teacher are...		
	No harm	The teacher and students survived in the tsunami meet a seriously injured man. There is some space left on their small boat. In order to send more people to a safe place as soon as possible, the teacher takes some actions. Eventually, the children are rescued but the injured man dies. The actions taken by the teacher are...	Saving	The man
20	Doing harm	An expedition is chased for breaking the taboo of the natives on an island. Sacrificing one person is the only way to abate natives' rage. In order to get more people rescued, the captain takes some actions on the seriously injured translator. Eventually, other people successfully escape, but the translator dies. The actions taken by the captain are ...	Handing over	The translator
	Allowing harm	An expedition is chased for breaking the taboo of the natives on an island. Sacrificing one person is the only way to abate natives' rage. In order to get more people rescued, the captain takes some actions on the translator who is left behind. Eventually, other people successfully escape, but the translator dies. The actions taken by the captain are ...	Giving up	The translator
	No harm	An expedition is chased for breaking the taboo of the natives on an island. Sacrificing one person is the only way to abate natives' rage. In order to save more people, the captain takes some actions on the seriously injured translator. Eventually, other people successfully escape, but the translator dies. The actions taken by the captain are ...	Carrying	The translator
21	Doing harm	A sudden failure causes a helicopter loaded with many tourists going to crash. The number of parachute is one less than the number of people on the helicopter. In order to let more people escape, the pilot takes some actions on the tourist who has an heart attack . Eventually, other tourists are saved but the tourist with heart attack dies. The actions taken by the pilot are...	Taking away	The parachute bag

	Allowing harm	A sudden failure causes a helicopter loaded with many tourists going to crash. There is no enough time to instruct all people how to use the parachute. In order to let more people escape, the pilot takes some actions on the tourist with a heart attack. Eventually, other tourists are saved but the tourist with a heart attack dies. The actions taken by the pilot are...	Giving up	The instruction
	No harm	A sudden failure causes a helicopter loaded with many tourists going to crash. At this moment, a tourist is extremely afraid of skydiving. In order to let more people escape, the pilot takes some actions on the tourist afraid of skydiving. Eventually, other tourists are saved but the tourist who is afraid of skydiving dies. The actions taken by the pilot are...	Encouraging	That tourist
22	Doing harm	When an expedition team is investigating in a mine, a collapse happens accidentally. Six team members are trapped in the mine, and the oxygen tank is about to run out. To get more people survive until the rescue, the captain takes some actions on a seriously injured team member who is in coma. Eventually, others are saved but the seriously injured team member dies. The actions taken by the captain are ...	Taking away	Oxygen
	Allowing harm	When an expedition team is investigating in a mine, a collapse happens accidentally. Six team members are trapped in the mine, and the second collapse is about to occur. To get more people survive until the rescue, the captain takes some actions on the seriously injured team member who is in coma. Eventually, others are saved but the seriously injured team member dies. The actions taken by the captain are ...	Leaving	That partner
	No harm	When an expedition team is investigating in a mine, a collapse happens accidentally. Six team members are trapped in the mine, and the rescue team is coming. To get more people survive until the rescue, the captain takes some actions on the seriously injured team member who is in coma. Eventually, others	Taking care of	That partner

		are saved but the seriously injured team member dies. The actions taken by the captain are ...		
23	Doing harm	A baby is diagnosed with a terminal illness and it causes great pain. In order not to make the baby suffer, the mother takes some actions. Eventually the baby dies. The actions taken by the mother are ...	Drowning	The baby
	Allowing harm	A baby is diagnosed with a terminal illness and the treatment is very painful. In order not to make the baby suffer, the mother takes some actions. Eventually the baby dies. The actions taken by the mother are …	Giving up	Therapy
	No harm	A baby is diagnosed with an incurable illness. In order not to make the baby suffer, the mother takes some actions. Eventually the baby dies. The actions taken by the mother are …	Looking for	A famous doctor
24	Doing harm	The doctor diagnoses a baby with a rare disease which cannot be cured and is very painful. In order not to the make the baby suffer, his father takes some actions. Eventually, the baby dies. The actions taken by the father are ...	Dashing	The baby
	Allowing harm	The doctor diagnoses a baby with a rare disease. The operation has little chance to be successful. In order not to make the baby suffer, his father takes some actions. Eventually, the baby dies. The actions taken by the father are ...	Giving up	Therapy
	No harm	The doctor diagnoses a baby with a rare disease. There is little chance of recovery. In order not to make the baby suffer, his father takes some actions. Eventually, the baby dies. The actions taken by the father are ...	Searching for	The famous doctor
25	Doing harm	The caravan is attacked by a lion when taking a rest on the prairie. A companion is seriously injured and bleeding. In order to divert the lion away from the caravan and get more people to the train to escape, the captain takes some actions. Eventually, the other people safely flee away , but the injured companion dies. The actions taken by the captain are ...	Pushing down	The companion
	Allowing harm	The caravan is attacked by a lion when taking a rest on the prairie. A companion	Ignoring	The companion

		is seriously injured and falls down from the truck. In order to divert the lion away from the caravan and get more people to the train to escape, the captain takes some actions. Eventually, the other people flee away safely, but the injured companion dies. The actions taken by the captain are ...		
	No harm	The caravan is attacked by a lion when taking a rest on the praire. A companion is attacked and falls down from the truck. In order to divert the lion away from the caravan and get more people to the train to escape, the captain takes some actions. Eventually, the other people flee away safely, but the injured companion dies. The actions taken by the captain are ...	Rescuing	The companion
26	Doing harm	An expedition team is attacked by wolves in the desert. A companion is seriously injured and bleeding. In order to divert the wolves away and get more people on the truck to escape, the leader takes some actions. Eventually, the other people flee away safely, but the injured companion dies. The actions taken by the leader are...	Pushing down	The companion
	Allowing harm	An expedition team is attacked by wolves in the desert. A companion is seriously injured and falls down from the truck. In order to divert the wolves away and get more people on the truck to escape, the leader takes some actions. Eventually, the other people flee away safely, but the injured companion dies. The actions taken by the captain are...	Ignore	The companion
	No harm	An expedition team is attacked by wolves in the desert.. A companion is attacked and falls down from the truck. In order to divert the wolves away and get more people on the truck to escape, the captain takes some actions. Eventually, the other people flee away safely, but the injured companion dies. The actions taken by the captain are...	Rescuing	The companion
27	Doing harm	Drug prices rise sharply after a deadly epidemic spreads. The pharmacy accidently catches fire at this moment. In order to take drugs which are used to	Trapping	The doctor

		save patients, the assistant takes some actions on the doctor who is trying to escape from the fire. Eventually, most patients are saved, but the doctor dies. The actions taken by the assistant on the doctor are ...		
	Allowing harm	Drug prices rise sharply after a deadly epidemic spreads. The pharmacy accidently catches fire at this moment. In order to save drugs which are used to save patients from the fire , the assistant takes some actions on the doctor who is trapped in the fire. Eventually, most patients are saved, but the doctor dies. The actions taken by the assistant on the doctor are ...	Giving up	The doctor
	No harm	Drug prices rise sharply after a deadly epidemic spreads. The pharmacy accidently catches fire at this moment. In order to let the doctor lower the price to save patients, the assistant takes some actions on the doctor who is trying to escape. Eventually, most patients are saved, but the doctor dies. The actions taken by the assistant on the doctor are ...	Rescuing	The doctor
28	Doing harm	A geological expedition team accidentally hangs from the cliff on a vine. A team member is physically tired. In order to prevent the vine from being broken which may lead to the death of the whole expedition, the team leader takes some actions on that member. Eventually, the other people are safe, but that member falls from the cliff and dies. The actions taken by the team leader are ...	Kicking down	The member
	Allowing harm	A geological expedition team accidentally hangs from the cliff on a vine. A team member is going to slide down. In order to prevent the vine from being broken which may lead to the death of the whole expedition, the team leader takes some actions on that member. Eventually, the other people are safe, but that member falls from the cliff and dies. The actions taken by the team leader are ...	Ignoring	The member
	No harm	A geological expedition team accidentally hangs from the cliff on a vine. A team member is trying to climb up to the cliff. In order to prevent the vine from being broken which may lead to the death of the whole expedition, the team leader takes	Assisting	The member

		some action on that member. Eventually, the other people are safe, but that member falls from the cliff and dies. The actions taken by the team leader are ...		
29	Doing harm	A rock climbing team hangs from the cliff accidently only depending on a rope. A member is physically tired. In order to prevent the rope from being broken which may lead to the death of all team members, the team leader takes some actions on that member. Eventually, the other people are safe, but that member falls from the cliff and dies. The actions taken by the team leader are ...	Kicking down	The member
	Allowing harm	A rock climbing team hangs from the cliff accidently only depending on a rope. A team member is going to slide down. In order to prevent the rope from being broken which may lead to the death of all team members, the team leader takes some actions on that member. Eventually, the other people are safe, but that member falls from the cliff and dies. The actions taken by the team leader are ...	Ignoring	The member
	No harm	A rock climbing team hangs from the cliff accidently only depending on a rope A team member is trying to climb up to the cliff. In order to prevent the rope from being broken which may lead to the death of all the team members, the team leader takes some actions on that member. Eventually, the other people are safe, but that member falls from the cliff and dies. The actions taken by the team leader are ...	Assisting	The member
30	Doing harm	A police car which escorts a serious criminal is accidentally trapped in the desert for several days. In order to save the lives of exhausted colleagues, the sheriff takes some actions. Eventually, the sheriff's colleagues are saved but the criminal dies. The actions taken by the sheriff are ...	Eating	The criminal
	Allowing harm	A police car which escorts a serious criminal is accidentally trapped in the desert for several days. In order to find a way to get out of the desert with the exhausted colleagues, the sheriff takes some actions. Eventually, the sheriff's colleagues are saved but the criminal dies. The actions taken by the sheriff are …	Leaving behind	The criminal

	No harm	A police car which escorts a serious criminal is accidentally trapped in the desert for several days. In order to get the criminal's help in repairing the equipment to get out of the desert, the sheriff takes some actions. Eventually, the sheriff's colleagues are saved but the criminal dies. The actions taken by the sheriff are …	Uncuffing	The criminal
31	Doing harm	Two policemen including a sheriff are attacked by a beast after they have captured a criminal in the mountain. As a result, a policeman is wounded. The sheriff takes some actions to distract the beast and save the life of his colleague, Eventually, the sheriff's colleague is saved but the criminal dies. The actions taken by the sheriff are ...	Cutting down	The criminal
	Allowing harm	Two policemen including a sheriff are attacked by a beast after they have captured a criminal in the mountain. As a result, a policeman is wounded. The sheriff takes some actions to save the life of his colleague in time, the. Eventually, the sheriff's colleague is saved but the criminal dies. The actions taken by the sheriff are ...	Leaving behind	The criminal
	No harm	Two policemen including a sheriff are attacked by a beast after they have captured a criminal in the mountain. As a result, a policeman is wounded. The sheriff takes some actions to beat back the beast to save the life of his colleague. Eventually, the sheriff's colleague is saved but the criminal dies. The actions taken by the sheriff are ...	Uncuffing	The criminal
32	Doing harm	Several mountain road-builders are trapped in a cave for several days without any food because of mudslides. In order to save more lives, the team leader takes some actions on a companion who is excessively bleeding. Eventually, other road-builders are saved but the excessively bleeding companion dies. The actions taken by the team leader are ...	Eating	The companion
	Allowing harm	Several mountain road-builders are trapped in a cave for several days without any food because of mudslides. In order to get more companions out of the cave	Leaving behind	The companion

		which is going to collapse, the team leader takes some actions on the companion who is excessively bleeding. Eventually, other road-builders are saved but the excessively bleeding companion died. The actions taken by the team leader are ...		
	No harm	Several mountain road-builders are trapped in a cave for several days without any food because of mudslides. In order to find ways to get more companions out of the cave , the captain takes some actions on the companion who is excessively bleeding. Eventually, other road-builders are saved but the excessively bleeding companion dies. The actions taken by the team leader are ...	Carrying	The companion
33	Doing harm	Several climbers are trapped below a cliff for several days because of landslides. Under the circumstance of no food, in order to save the lives of most people, the team leader takes some actions on a dying companion. Eventually, other climbers are survived, but that dying companion dies. The actions taken by the team leader on that companion are ...	Eating	The companion
	Allowing harm	Several climbers are trapped below a cliff for several days because of landslides. Under the circumstance of no help, in order to save the lives of most people, the team leader takes some actions on a dying companion. Eventually, other climbers are survived, but that dying companion dies. The actions taken by the team leader on that companion are ...	Leaving behind	The companion
	No harm	Several climbers are trapped below a cliff for several days because of landslides. Luckily, the food and water are adequate temporarily. In order to save the lives of most people, the team leader takes some actions on a seriously injured companion. Eventually, other climbers are survived, but the seriously injured companion dies. The actions taken by the team leader on the seriously injured companion are ...	Taking care of	The companion
34	Doing harm	A serious fault happens to a sightseeing submarine. A tourist suddenly becomes psychotic and seriously disrupts the order of escaping. In order to get most	Stunning	The tourist

		tourists escape in time, the person in charge takes some actions on the psychotic tourist. Eventually, other tourists successfully escape, but the psychotic tourist dies. The actions taken by the person in charge are ...		
	Allowing harm	A serious fault happens to a sightseeing submarine. A tourist suddenly becomes psychotic and strongly rejects to escape. In order to get most tourists escape in time, the person in charge takes some actions on the psychotic tourist. Eventually, other tourists successfully escape, but the psychotic tourist dies. The actions taken by the person in charge are ...	Giving up	The tourist
	No harm	A serious fault happens to a sightseeing submarine. A tourist suddenly becomes psychotic and seriously disrupts the order of escaping. In order to get most tourists escape in time, the person in charge takes some actions on the psychotic tourist. Eventually, other tourists successfully escape, but the tourist dies. The actions taken by the person in charge are ...	Pacifying	The tourist
35	Doing harm	Several weapon experts are tortured by the terrorists. A colleague cannot bear the torture and is going to confess. In order to keep the secret information, the group leader takes some actions. Eventually, the secret information is preserved but that colleague dies. The actions taken by the group leader are ...	Poisoning	The colleague
	Allowing harm	Several weapon experts are tortured by the terrorists. A colleague cannot bear the torture and is going to commit suicide. In order to keep the secret information, the group leader takes some actions. Eventually, the secret information is preserved but that colleague dies. The actions taken by the group leader are ...	Allowing to	Commit suicide
	No harm	Several weapon experts are tortured by the terrorists. A colleague cannot bear the torture and is in hesitation about whether to confess. In order to keep the secret information, the group leader takes some actions. Eventually, the secret information is preserved but that colleague dies. The actions taken by the group leader are ...	Persuading	The colleague

36	Doing harm	A small tractor that is sliding down the ramp and running towards several children who are playing downhill. At this moment, a big blind man is passing by. In order to change the direction of the running tractor to save the lives of the children, a cleaner takes some actions on the big blind man. Eventually, the children are saved but the tractor kills the blind man. The actions taken by the cleaner are ...	Pushing	The blind man
	Allowing harm	A small tractor that is sliding down the ramp and running towards several children who are playing downhill. At this moment, a big blind man is walking to the tractor. In order to change the direction of the running tractor to save the lives of the children, a cleaner takes some actions on the big blind man. Eventually, the children are saved but the tractor kills the blind man. The actions taken by the cleaner are ...	Ignoring	The blind man
	No harm	A small tractor that is sliding down the ramp and running towards several children who are playing downhill. At this moment, a big blind man keenly detects what is happening and trying to stop the sliding tractor. In order to save the lives of the children, a cleaner takes some actions on the big blind man. Eventually, the children are saved but the tractor kills the blind man. The actions taken by the cleaner are ...	Assisting	The blind man
37	Doing harm	After a major earthquake, the hospital is desperately short of medicine. An enemy of the medicine businessman is ill and coming to ask the hospital president for prescription. In order to save more lives, the hospital president takes some actions on this patient. Eventually, the hospital president obtains a large amount of medicine for the hospital, but the patient dies. The actions taken by the hospital president are …	Poisoning	The patient
	Allowing harm	After a major earthquake, the hospital is desperately short of medicine. An enemy of the medicine businessman comes to the hospital to have a surgery. In order to	Giving up	The surgery

		save more lives, the hospital president takes some actions on this patient. Eventually, the hospital president obtains a large amount of medicine for the hospital, but the patient dies. The actions taken by the hospital president are …		
	No harm	After a major earthquake, the hospital is desperately short of medicine. An enemy of the medicine businessman comes to visit the hospital. In order to save more lives, the hospital president takes some actions on the enemy. Eventually, the hospital president obtains a large amount of medicine for the hospital, but the enemy dies. The actions taken by the hospital president are …	Solving	The contradiction
38	Doing harm	A seriously wounded soldier escapes to a small village for rehabilitation, but is found by the pursuing forces. In order to prevent the forces venting anger on the village and killing the villagers, the village head takes some actions on the wounded soldier. Eventually, the villagers are safe but the soldier dies. The actions taken by the village head are...	Killing	The soldier
	Allowing harm	A seriously wounded soldier escapes to a small village for rehabilitation, but the pursuing forces are coming. In order to prevent the forces venting anger on the village and killing the villagers, the village head takes some actions on the wounded soldier. Eventually, the villagers are safe but the soldier dies. The actions taken by the village head are...	Giving up	The treatment
	No harm	A seriously wounded soldier escapes to a small village for rehabilitation, but the pursuing forces are coming. In order to prevent the forces venting anger on the village and killing the villagers, the village head takes some actions on the wounded soldier. Eventually, the villagers are safe but the soldier dies. The actions taken by the village head are...	Escorting	The soldier
39	Doing harm	Several workers are trapped in a mine. Everyone is exhausted. In order to save most people's lives, the team leader takes some actions on the worker who is excessively bleeding. Eventually, other people are saved, but the bleeding worker	Eating	The worker

		dies. The actions taken by the team leader are ...		
	Allowing harm	Several workers are trapped in a mine. Everyone is exhausted. In order to save most people's lives before the next collapse, the team leader takes some actions on the workers who is excessively bleeding. Eventually, other people are saved, but the bleeding worker dies. The actions taken by the captain are ...	Giving up	The worker
	No harm	Several workers are trapped in a mine. Everyone is exhausted. In order to find ways out to save most people's lives, the team leader takes some actions on the workers who is excessively bleeding. Eventually, other people are saved, but the bleeding worker dies. The actions taken by the team leader are ...	Carrying	The worker
40	Doing harm	A businessman insists on taking back the house property originally used by the orphanage. A heart attack happens to him when he is negotiating with the orphanage director, and the medicine bottle accidentally falls to the ground. In order to avoid the children's displacement, the director takes some actions. Eventually the children are able to continue to live in the orphanage, but the businessman dies. The actions taken by the director are...	Kicking off	The medicine bottle
	Allowing harm	A businessman insists on taking back the house property originally used by the orphanage. A heart attack happens to him when he is negotiating with the orphanage director. In order to avoid the children's displacement, the director takes some actions . Eventually the children are able to continue to live in the orphanage, but the businessman dies. The actions taken by the director are...	Ignoring	The businessman
	No harm	A businessman insists on taking back the house property originally used by the orphanage. A heart attack happens to him when he is negotiating with the orphanage director. In order to make the businessman change his decision, the director takes some actions. Eventually the children are able to continue to live in the orphanage, but the businessman dies. The actions taken by the director are...	Helping	The businessman
41	Doing harm	A military team on a secret mission encounters a wounded civilian who doesn't	Killing	The civilian

		draw a clear distinction between the enemy and us. In order to avoid the news get out and threaten the life of the team members, the leader takes some actions. Eventually, the military team completes the task safely, but the civilian dies. The actions taken by the leader are ...		
	Allowing harm	A military team on a secret mission encounters a wounded civilian who doesn't draw a clear distinction between the enemy and us. In order to complete the task in time and ensure the safety of the team members, the leader takes some actions . Eventually, the military team completes the task safely, but the civilian dies. The actions taken by the leader are ...	Giving up	The civilian
	No harm	A military team on a secret mission encounters a wounded civilian who doesn't draw a clear distinction between the enemy and us. In order to inquire about the local news to promote the completion of the task, the leader takes some actions. Eventually, the military team completes the task safely, but the civilian dies. The actions taken by the leader are ...	Saving	The civilian
42	Doing harm	Several skiers encounter a snow slide and it is hard for helicopters to find survivors in the white snow. It seems that only the blood can attract the rescue. In order to get more people rescued, the leader takes some actions on the injured companion. Eventually, other people are rescued, but the injured companion dies. The actions taken by the leader are ...	Killing	The companion
	Allowing harm	Several skiers encounter a snow slide and it is hard for helicopters to find survivors in the white snow. They don't have any physical strength to take care of the wounded companion. In order to get more people rescued, the leader takes some actions on the injured companion. Eventually, other people are rescued, but the injured companion dies. The actions taken by the leader are ...	Giving up	The companion
	No harm	Several skiers encounter a snow slide and it is hard for helicopters to find survivors in the white snow. The injured companion cannot hold on. In order to	Caring for	The companion

		get more people rescued, the leader takes some actions on the injured companion. Eventually, other people are rescued, but the injured companion dies. The actions taken by the leader are ...		
43	Doing harm	An isolated village encounters successive years of famine. A large number of villagers starve to death but no place to escape. In order to save the lives of young, the village head takes some actions on the frail old people. Eventually, the young people go through the famine years, but most old people die. The actions taken by the village head are ...	Eating	The old people
	Allowing harm	A remote village encounters successive years of famine. Young people consider going out to survive but are unable to take the old people. In order to make more people survive, the village head takes some actions. Eventually, the young people go through the famine years, but most old people die. The actions taken by the village head are ...	Leaving	The old people
	No harm	A remote village encounters successive years of famine. Old people are weak but willing to follow the young people to go out to survive. In order to make more people survive, the village head takes some actions. Eventually, the young people go through the famine years, but most old people die. The actions taken by the village head are ...	Taking along	The old people
44	Doing harm	A cruise ship is about to sink after hitting the rocks. The number of the lifeboat is limited. A man tries to forcibly board a lifeboat and seriously disrupts the escape order. In order to make more people escape orderly, the captain takes some actions. Eventually, most people are rescued, but the man dies. The actions taken by the captain are ...	Shooting	The man
	Allowing harm	A cruise ship is about to sink after hitting the rocks. The number of the lifeboat is limited. A man crashes into the sea when forcibly boarding the lifeboat. In order to make more people escape orderly, the captain takes some actions. Eventually,	Disregarding	The man

		most people are rescued, but the man dies. The actions taken by the captain are ...		
	No harm	A cruise ship is about to sink after hitting the rocks. The number of the lifeboat is limited. A man voluntarily helps women and children board the lifeboat firstly. In order to make more people escape orderly, the captain takes some actions. Eventually, most people are rescued, but the man dies. The actions taken by the captain are ...	Helping	The man
45	Doing harm	A gangster with a knife who is equipped with the explosives hijacks a bus. If someone can knock down the gangster, the driver will have the opportunity to subdue him. In order to ensure most passengers' safety, the driver takes some actions on the man behind him. Eventually, the other passengers successfully escape, but that man is dying. The actions taken by the driver are ...	Thrusting	The man
	Allowing harm	A gangster with a knife who is equipped with the explosives hijacks a bus. After arriving at the destination, the gangster hijacks a man as a hostage. In order to ensure most passengers' safety, the driver takes some actions on the man who is hijacked. Eventually, the other passengers successfully escape, but that man is dying. The actions taken by the driver are ...	Disregarding	The man
	No harm	A gangster with a knife who is equipped with the explosives hijacks a bus. A man is trying to subdue the gangster. In order to ensure most passengers' safety, the driver takes some actions on that man. Eventually, other passengers successfully escape, but that man is dying. The actions taken by the driver are ...	Assisting	The man
46	Doing harm	A man finds that his son is born because his wife commits an adultery. Thus, he holds a knife and rushes to the kindergarten to find his son. In order to protect more children, the teacher takes some actions on the boy. Eventually, other children are not harmed, but the boy is seriously injured. The actions taken by the teacher are ...	Handing over	The boy
	Allowing harm	A man finds that his son is born because his wife commits an adultery. Thus, he	Giving up	The boy

		holds a knife and rushes to the kindergarten to take away his son. In order to protect more children, the teacher takes some actions on the boy. Eventually, other children are not harmed, but the boy is seriously injured. The actions taken by the teacher are ...		
	No harm	A man finds that his son is born because his wife commits an adultery. Thus, he holds a knife and rushes to the kindergarten. In order to protect more children, the teacher takes some actions on the boy after sending other children. Eventually, other children are not harmed, but the boy is seriously injured. The actions taken by the teacher are ...	Protecting	The boy
47	Doing harm	A rubber boat strays from its proper course and is trapped in the rapids under the dam, and a tourist’s vest is washed away. In order to save more lives, the tour guide takes some actions on the terminally-ill tourist. Eventually, other people are saved but the terminally-ill tourist dies. The actions taken by the tour guide are ...	Taking away	The vest
	Allowing harm	A rubber boat strays from its proper course and is trapped in the rapids under the dam, but the tour guide cannot get all the people escape at the same time. In order to save more lives, the tour guide takes some actions on the terminal-ill tourist. Eventually, other people are saved but the terminal-ill tourist dies. The actions taken by the tour guide are ...	Giving up	The tourist
	No harm	A rubber boat trays from its proper course and is trapped in the rapids under the dam, and a tourist suddenly has an asthmatic attack. In order to save more lives, the tour guide takes some actions on the tourist with asthma. Eventually, other people are saved but the terminal-ill tourist dies. The actions taken by the tour guide are ...	Helping	The tourist
48	Doing harm	Six pacifists are held as hostages by terrorists, when they visiting a war-torn country. In order to get the trust of terrorists to save more people, the translator takes some actions on a hostage with serious injury. Eventually, other people are	Killing	The hostage

		saved but that injured hostage dies. The actions taken by the translator are ...		
	Allowing harm	Six pacifists are held as hostages by terrorists, when they visiting a war-torn country. In order to seize the rare opportunity to save more people in time, the translator takes some actions on a hostage with serious injury. Eventually, other people are saved but that injured hostage dies. The actions taken by the translator are ...	Giving up	The hostage
	No harm	Six pacifists are held as hostages by terrorists, when they visiting a war-torn country. In order to let the hostages hold on until help comes, the translator takes some actions on a hostage with serious injury. Eventually, other people are saved but that injured hostage dies. The actions taken by the translator are ...	Helping	The hostage
49	Doing harm	A girl and an old man who both survive in an airplane crash and get on an island suffer from disease attacks at the same time. They almost run out of the drugs and the old man cannot endure the pain any more. In order to save the girl's life, the nurse takes some actions on the sick old man. Eventually, the girl is saved but the old man dies. The actions taken by the nurse are ...	Smothering	The old man
	Allowing harm	A girl and an old man who both survive in an airplane crash and get on an island suffer from disease attacks at the same time. They almost run out of the drugs and the old man has multiple injuries. In order to save the girl's life, the nurse takes some actions on the sick old man. Eventually, the girl is saved but the old man dies. The actions taken by the nurse are ...	Giving up	The old man
	No harm	A girl and an old man who both survive in an airplane crash and get on an island suffer from disease attacks at the same time. They almost run out of the drugs but there are alternative herbal medicines on the island. In order to save the patients, the nurse takes some actions on both the girl and the old man. Eventually, the girl is saved but the old man dies. The actions taken by the nurse are ...	Treating	Two patients
50	Doing harm	A doctor without borders receives a pregnant woman in urgent need of blood	Cutting off	The blood

		transfusion, when he is giving a disabled blood transfusion in the war. In order to save the lives of pregnant woman and the unborn baby, the doctor takes some actions. Eventually, the pregnant woman is saved but the disabled dies. The actions taken by the doctor are ...		transfusion
	Allowing harm	A doctor without borders receives a pregnant woman in instant need of first aid, when he is treating a seriously injured disabled in the war. In order to save the lives of the pregnant woman and the unborn baby, the doctor takes some actions. Eventually, the pregnant woman is saved but the disabled dies. The actions taken by the doctor are ...	Giving up	The treatment
	No harm	When a doctor without borders rescues a seriously injured disabled and a pregnant woman in the war, his colleagues arrived in time. In order to save the lives of pregnant woman and the unborn baby, the doctor takes some actions. Eventually, the pregnant woman is saved but the disabled dies. The actions taken by the doctor are ...	Requesting	Help
51	Doing harm	An old man is entangled with aquatic weeds in the water when swimming. He instinctively grasps the child next to him. The old man clutches the child and don’t let him go. In order to save the child's life, the administrator takes some actions. Eventually, the child is saved but the old man dies. The actions taken by the administrator are ...	Knocking out	The old man
	Allowing harm	An old man is entangled with aquatic weeds in the water when swimming. He instinctively grasps the child next to him. The administrator cannot save both of them at the same time. In order to save the child's life, the administrator takes some actions. Eventually, the child is saved but the old man dies. The actions taken by the administrator are ...	Giving up	The old man
	No harm	An old man is entangled with aquatic weeds in the water when swimming. He instinctively grasps the child next to him. The administrator immediately	Saving	The two persons

		discovers the situation. In order to save their lives, the administrator takes some actions. Eventually, the child is saved but the old man dies. The actions taken by the administrator are ...		
52	Doing harm	When a tour bus driving past a wooden bridge, the driver finds the bridge is going to collapse. A tour guide falls down and is hard to stand up on the deck in the front. If the driver brakes at this time, all passengers will be in danger. For the safety of more people, the driver takes some actions. Eventually, all passengers are safe, but the tour guide dies. The actions taken by the driver are ...	Running over	The tour guide
	Allowing harm	When a tour bus driving past a wooden bridge, the driver finds the bridge is going to collapse. A tour guide is walking on the deck in the front. If the driver stops the bus and saves the tour guide at this time, all passengers will be in danger. For the safety of more people, the driver takes some actions. Eventually, all passengers are safe, but the tour guide dies. The actions taken by the driver are ...	Ignoring	The tour guide
	No harm	When a tour bus driving past a wooden bridge, the driver finds the bridge is going to collapse. A tour guide is crossing the bridge from the other side. For the safety of more people, the driver takes some actions. Eventually, all passengers are safe, but the tour guide dies. The actions taken by the driver are ...	Reminding	The tour guide
53	Doing harm	Terrorists bind a time bomb on a hostage and threaten him to go to the city center. The rescue team cannot arrive in time. In order to prevent the bomb explosion in a crowded place, a policeman takes some actions. Eventually, no casualties around, but the hostage dies. The actions taken by the policeman are ...	Detonating	The bomb
	Allowing harm	Terrorists bind a time bomb on a hostage and threaten him to go to the city center. The rescue team cannot arrive in time. In order to seize the time to disperse the crowd before the bomb explosion, a policeman takes some actions. Eventually, no casualties around, but the hostage dies. The actions taken by the policeman are ...	Giving up	The hostage
	No harm	Terrorists bind a time bomb on a hostage and threaten him to go to the city center.	Rescuing	The hostage

		The rescue team cannot arrive in time. In order to ensure the safety of the hostage and the public, a policeman takes some actions. Eventually, no casualties around, but the hostage dies. The actions taken by the police are ...		
54	Doing harm	The circus lion is frightened and jumps off the stage when performing jumping through the fire tightropes. At this time, the lion's attention must be drawn away as soon as possible; otherwise the audience is very dangerous. For the safety of the audience, the trainer takes some actions. Eventually, the audience is safe, but the assistant loses an arm. The actions taken by the trainer are ...	Pushing down	The assistant
	Allowing harm	The circus lion is frightened and jumps on the assistant when performing jumping through the fire tightropes. In order to evacuate the audience as soon as possible to ensure everyone’s safety, the trainer takes some actions on the assistant. Eventually, the audience is safe, but the assistant loses an arm. The actions taken by the trainer are ...	Ignoring	The assistant
	No harm	The circus lion is frightened while performing jumping through the fire tightropes, and the assistant tries to control the lion. For the safety of the audience and the assistant, the trainer takes some actions on the assistant. Eventually, the audience is safe, but the assistant loses an arm. The actions taken by the trainer are ...	Helping	The assistant
55	Doing harm	A murderer who evaded justice is taken to the hospital because of gas poisoning. After treatment and his life is saved, the doctor learns that the murderer is still planning to hurt other people. To avoid more innocent lives being hurt, the doctor takes some actions on the murderer. Eventually, the innocent lives are saved but the murderer dies. The actions taken by the doctor are ...	Cutting off	The oxygen infusion
	Allowing harm	A murderer who evaded justice is taken to the hospital because of gas poisoning. Before treatment, the doctor learns that the murderer is still planning to hurt other people. To avoid more innocent lives being hurt, the doctor takes some actions on	Giving up	The rescue

		the murderer. Eventually, the innocent lives are saved but the murderer dies. The actions taken by the doctor are ...		
	No harm	A murderer who evaded justice is taken to the hospital because of gas poisoning. During the recovery phase, the doctor learned that the killer is still planning to hurt other people. To avoid more innocent lives being hurt, the doctor takes some actions on the murderer. Eventually, the innocent lives are saved but the murderer dies. The actions taken by the doctor are ...	Persuading	The murderer
56	Doing harm	A wooden handcart loading with woods is sliding down the ramp and running towards several loggers. At this moment, a big fat man is passing by. In order to change the direction of the running handcart to save more lives, the foreman takes some actions on the big fat man. Eventually, all workmates are saved but the big fat man is killed by the handcart. The actions taken by the foreman are ...	Pushing down	The fat man
	Allowing harm	A wooden handcart loading with woods is sliding down the ramp and running towards several loggers. At this moment, a big fat man bowing to play the cell phone is walking to the wooden handcart. In order to change the direction of the running handcart to save more lives, the foreman takes some actions on the big fat man. Eventually, all workmates are saved but the big fat man is killed by the handcart. The actions taken by the foreman are ...	Ignoring	The fat man
	No harm	A wooden handcart loading with woods is sliding down the ramp and running towards several loggers. At this moment, a big fat man is trying to stop the handcart. In order to save the lives of workmates, the foreman takes some actions on the big fat man. Eventually, all workmates are saved but the big fat man is killed by the handcart. The actions taken by the foreman are ...	Helping	The fat man
57	Doing harm	A child accidentally runs into the medical laboratory, and triggers the toxic gas. The laboratory does not have an extra gas mask. In order to save the child’s life, the person in charge takes some actions on a terminal-ill colleague. Eventually,	Taking away	The mask

		the toxic gas is under control, but the colleague dies. The actions taken by the person in charge are ...		
	Allowing harm	A child accidentally runs into the medical laboratory, and triggers the toxic gas. The person in charge cannot save the child and the fainted colleague at the same time. In order to save the child's life, the person in charge takes some actions on the colleague. Eventually, the toxic gas is under control, but the colleague dies. The actions taken by the person in charge are ...	Giving up	The colleague
	No harm	A child accidentally runs into the medical laboratory, and triggers the toxic gas. Someone must stay to take care of it. In order to save more lives, the person in charge takes some actions on the colleague who adheres to stay. Eventually, the toxic gas is under control, but the colleague dies. The actions taken by the person in charge are ...	Helping	The colleague
58	Doing harm	A family of four inadvertently witnessing a murder is chased by the gangster with knives. If they do not try to pin down the gangster, it is difficult to escape. In order to take the children over the wall to escape, the man takes some actions on his wife who hurts feet. Eventually, the man takes the children out of danger, but the wife dies. The actions taken by the man are ...	Pushing down	His wife
	Allowing harm	A family of four inadvertently witnessing a murder is chased by the gangster with knives. If taking his wife who hurts feet along with them, it is difficult for children and him to escape. In order to take the children over the wall to escape, the man takes some actions on his wife. Eventually, the man takes the children out of danger, but his wife dies. The actions taken by the man are ...	Leaving behind	His wife
	No harm	A family of four inadvertently witnessing a murder is chased by the gangster with knives. If they want to get rid of the gangster, they could only attempt to climb over the wall. In order to make the children escape, the man take some actions on his wife who is carrying the children onto the wall. Eventually, the man takes the	Helping	His wife

		children out of danger, but the wife dies. The actions taken by the man are ...		
59	Doing harm	A residential building is on fire, an old woman with psychiatric disorders and her grandson are still inside. The old woman holds the child tightly and refuses to escape. In order to save the child's life, a neighbor takes some actions. Eventually, the child is saved but the old woman dies. The actions taken by the neighbor are ...	Knocking out	The old woman
	Allowing harm	A residential building is on fire, an old woman with psychiatric disorders and her grandson are still inside. A neighbor cannot save both of the old woman and her grandson at the same time. In order to save the child's life, the neighbor takes some actions. Eventually, the child is saved but the old woman dies. The actions taken by the neighbor are ...	Giving up	The old woman
	No harm	A residential building is on fire, an old woman with psychiatric disorders and her grandson are still inside. The old woman holds the child tightly and refuses to escape. In order to save their lives, a neighbor takes some actions. Eventually, the child is saved but the old woman dies. The actions taken by the neighbor are ...	Persuading	The old woman
60	Doing harm	A girl with cancer commits suicide for many times because of extreme despair. In order to avoid her suffering, her mother takes some actions. Eventually, the girl dies. The measures taken by her mother are ...	Poisoning	Her daughter
	Allowing harm	A girl with cancer commits suicide for many times because of unbearable chemotherapy. In order to alleviate the pain caused by the treatment, her mother takes some actions. Eventually, the girl dies. The actions taken by her mother are ...	Giving up	The treatment
	No harm	A girl with cancer commits suicide for many times because of unbearable pain. In order to make the girl strong and optimistic, her mother takes some actions. Eventually, the girl dies. The actions taken by her mother are ...	Encouraging	Her daughter

Non-moral dilemmas in English			
**No.**	**Scenario**	**Protagonist's action**	**Object of the protagonist's action**
1	It's unusually hot this summer, and shopping malls have a lot of sales promotion. In order to improve the comfort, Xiao Li takes some actions. Eventually, he spent the summer comfortably. The actions taken by Xiao Li are...	Installing	Air conditioner
2	A student often eats spicy food leading to gastrointestinal discomfort. In order to be in good health, the student takes some actions. Eventually, his intestines and stomach get better. The actions taken by the student are...	Getting rid of	The habit
3	In the market, the price of pork is frequently rising and the family loves to eat chicken. In order to reduce the household expenses, the housewife takes some actions. Eventually, the household expenses are reduced. The actions taken by the housewife are...	Buying	chicken
4	Self-study classroom is too hot. In order to make the air circulate, the senior takes some actions. Finally the classroom is no longer so hot. The actions taken by the senior are...	Opening	The window
5	The kid does not like vegetables. In order to ensure the kid's nutrition, his mother takes some actions. Finally, the kid loves to eat vegetables. The actions taken by his mother are...	Improving	Cooking skills
6	The elevator door is about to close, when an employee is arriving at the company downstairs. In order not to be late, the employee takes some actions. Eventually, he gets to the office at the given time. The actions taken by the employee are...	Catching up with	The elevator
7	The rainy season comes, there are puddles everywhere. In order to prevent the shoes getting wet, Xiao Wang takes some actions. Eventually, Xiao Wang's shoes are no	Preparing	Shoe covers

	longer wet. The actions taken by Xiao Wang are...		
8	This shopping mall lays in a new stock of aircraft models. In order to buy his favorite aircraft model, Xiao Wang takes some actions. Finally, he makes it. The actions taken by Xiao Wang are...	Saving	Money
9	The old man in the supermarket is going to buy soy sauce and towel, but he finds that he does not have enough money. In order not to delay to cook dinner, the old man takes some actions. Finally, he makes it. The actions taken by the elderly are...	Giving up	The towel
10	Xiao Wang gets a temporary business trip. In order to ensure the safety of family circuit, he takes some actions. Eventually, family circuit is alright. The actions taken by Xiao Wang are...	Turning off	Power supply
11	A young man gets trapped in a park because of a sudden heavy rain. In order to arrive before the movie begins, the man takes some actions. Eventually, he gets to the cinema in time. The actions taken by the man are...	Walking in	The rain
12	Laboratory computers often have problems. In order to improve the efficiency of scientific research, the teacher takes some actions. Finally, the efficiency of scientific research is improved. The actions taken by the teacher are...	Changing	Computers

Experimental moral dilemmas in Chinese				
**No.**	**Resolution type**	**Scenario**	**Protagonist's action**	**Object of the protagonist's action**
1	Doing harm	某急诊室医生在给一名重伤者输血时又接到五名轻伤者。轻伤者若不及时输血也会导致病情恶化而丧身，而此时血库告急。为了挽救更多的生命，医生对重伤者采取了一定的措施。最终五名轻伤者得救，重伤者死亡。医生采取的措施是……	切断	输血
	Allowing harm	某急诊室医生在给一名重伤者抢救时又接到五名轻伤者。轻伤者若不及时治疗也会导致病情恶化而丧身，而此时人手紧缺。为了挽救更多的生命，医生对重伤者采取了一定的措施。最终五名轻伤者得救，重伤者死亡。医生采取的措施是……	放弃	治疗
	No harm	某急诊室医生在给一名重伤者抢救时又接到五名轻伤者。轻伤者若不及时治疗也会导致病情恶化而丧身，而此时同事有空。为了挽救更多的生命，医生对该同事采取了一定的措施。最终五名轻伤者得救，重伤者死亡。医生采取的措施是……	请求	协助
2	Doing harm	某生物学家在实验中触发了一种极端危险的病毒。若不及时关闭安全门，会导致病毒扩散威胁更多人的生命。而此时生物学家还未逃出。为了及时关闭安全门，生物学家的学生采取了一定的措施。最终病毒得到了控制，生物学家死亡。学生采取的措施是……	关闭	大门
	Allowing harm	某生物学家在实验中触发了一种极端危险的病毒。若开门营救生物学家，会导致病毒扩散威胁更多人的生命。而此时生物学家已经晕倒。为了及时关闭安全门，生物学家的学生采取了一定的措施。最终病毒得到了控制，生物学家死亡。学生采取的措施是……	放弃	开门
	No harm	某生物学家在实验中触发了一种极端危险的病毒。若不及时关闭安全门，会导致病毒扩散威胁更多人的生命。而此时生物学家步履蹒跚。为了及时关闭安全门，生物学家的学生采取了一定的措施。最终病毒得到了控制，生物学家死亡。学生采取的措施是……	营救	老师

3	Doing harm	患有精神病的大儿子“阿牛”抱着自己的弟弟跳入河中。由于阿牛死死地抱着弟弟，父亲无法营救。为了不失去健康的小儿子，父亲采取了一定的措施。最终小儿子得救，阿牛死亡。父亲采取的措施是……	溺死	阿牛
	Allowing harm	患有精神病的大儿子“阿牛”抱着自己的弟弟跳入河中。由于水流非常湍急，父亲无法同时救起两人。为了不失去健康的小儿子，父亲采取了一定的措施。最终小儿子得救，阿牛死亡。父亲采取的措施是……	放弃	阿牛
	No harm	患有精神病的大儿子“阿牛”抱着自己的弟弟跳入河中。由于父亲离得不远，及时发现了这一情况。为了不失去自己的孩子们，父亲采取了一定的措施。最终小儿子得救，阿牛死亡。父亲采取的措施是……	救起	两人
4	Doing harm	某痴呆症老人和他的孙子被困火海。而老人死死地抱着孩子拒绝逃生。为了挽救孩子的生命，老人的儿子采取了一定的措施。最终孩子得救，老人死亡。老人的儿子采取的措施是……	打晕	父亲
	Allowing harm	某痴呆症老人和他的孙子被困火海。而老人的儿子无法同时救走两人。为了挽救孩子的生命，老人的儿子采取了一定的措施。最终孩子得救，老人死亡。老人的儿子采取的措施是……	放弃	父亲
	No harm	某痴呆症老人和他的孙子被困火海。而老人的儿子听说后立刻赶到。为了挽救亲人的生命，老人的儿子采取了一定的措施。最终孩子得救，老人死亡。老人的儿子采取的措施是……	背走	两人
5	Doing harm	村民们在躲避敌人时发现一个重病的婴儿，无论如何都无法让她止住啼哭。若不及时止住婴儿的哭声，所有人的生命都将受到威胁。为了防止哭声引来敌人，村长采取了一定的措施。最终村民们安全躲过敌人，婴儿死亡。村长采取的措施是……	捂死	婴儿
	Allowing harm	村民们在躲避敌人时发现一个重病的婴儿，无论如何都无法让她止住啼哭。若带上这个不停啼哭的婴儿，所有人的生命都将受到威胁。为了防止哭声引来敌人，村长采取了一定的措施。最终村民们安全躲过敌人，婴儿死亡。村长采取的措施是……	放弃	婴儿
	No harm	村民们在躲避敌人时发现一个重病的婴儿，只有村长的安抚能让她止住啼哭。若不及时止住婴儿的哭声，所有人的生命都将受到威胁。为了防止哭声引来敌人，村长	安抚	婴儿

		采取了一定的措施。最终村民们安全躲过敌人，婴儿死亡。村长采取的措施是……		
6	Doing harm	在躲避敌军时，一名新兵误中猎人的陷阱，严重受伤。此时敌人已经临近，若没有人滚下山坡引开敌人，所有人都会陷入险境。为了挽救更多士兵的生命，队长对该新兵采取了一定的措施。最终其他人安全躲过敌人，新兵死亡。队长对新兵采取的措施是……	推下	新兵
	Allowing harm	在躲避敌军时，一名新兵误中猎人的陷阱，严重受伤。此时敌人已经临近，若带上这位受伤的新兵逃离，所有人都会陷入险境。为了挽救更多士兵的生命，队长对该新兵采取了一定的措施。最终其他人安全躲过敌人，新兵死亡。队长对新兵采取的措施是……	放弃	新兵
	No harm	在躲避敌军时，一名新兵误中猎人的陷阱，严重受伤。此时敌人快要追来，没有足够时间带该新兵逃生，只允许快速将他隐藏。为了挽救更多士兵的生命，队长对该新兵采取了一定的措施。最终其他人安全躲过敌人，新兵死亡。队长对新兵采取的措施是……	隐藏	新兵
7	Doing harm	一艘渔船迷失在大海中多日而食物匮乏，其中一位船员已经虚弱不堪。若要继续坚持等待救援，只能吃掉同伴维持生命。为了挽救更多生命，船长对那名虚弱的船员采取了一定的措施。最终其他人得救，该船员死亡。船长对该船员采取的措施是……	吃掉	船员
	Allowing harm	一艘渔船迷失在大海中多日而食物匮乏，其中一位船员已经虚弱不堪。若要保存体力等待救援，便不能照顾病弱的同伴。为了挽救更多生命，船长对那名虚弱的船员采取了一定的措施。最终其他人得救，该船员死亡。船长对该船员采取的措施是……	不顾	船员
	No harm	一艘渔船迷失在大海中多日而食物匮乏，其中一位船员已经虚弱不堪。若要继续坚持等待救援，就不能削弱求生的意念。为了挽救更多生命，船长对那名病弱的船员采取了一定的措施。最终其他人得救，该船员死亡。船长对该船员采取的措施是……	鼓励	船员
8	Doing harm	船长和几名游客在海难中流落到没有食物的荒岛上，其中一名游客还受了重伤。大家若要继续生存，只能吃掉同伴维持生命。为了更多人的存活，船长对重伤的游客采取了一定的措施。最终其他人得救，该游客死亡。船长对该游客采取的措施是……	吃掉	游客
	Allowing harm	船长和几名游客在海难中流落到没有食物的荒岛上，其中一名游客还受了重伤。大	不顾	游客

		家若要继续生存，便不能花费额外的体力。为了更多人的存活，船长对重伤的游客采取了一定的措施。最终其他人得救，该游客死亡。船长对该游客采取的措施是……		
	No harm	船长和几名游客在海难中流落到没有食物的荒岛上，其中一名游客还受了重伤。大家若要坚持下来，就要保持求生的意念。为了更多人的存活，船长对重伤的游客采取了一定的措施。最终其他人得救，该游客死亡。船长对该游客采取的措施是……	鼓励	游客
9	Doing harm	刚将小女孩从洪水中救上木板的男子又遇到一位老人。木板太过脆弱，无法承受两人的重量。为了小女孩的生命安全，男子对老人抓住木板的手采取了一定的措施。最终小女孩得救，老人死亡。男子采取的措施是……	掰开	手指
	Allowing harm	刚将小女孩从洪水中救上木板的男子又遇到一位老人。木板太过脆弱，无法承受两人的重量。为了小女孩的生命安全，男子对即将溺水的老人采取了一定的措施。最终小女孩得救，老人死亡。男子采取的措施是……	放弃	老人
	No harm	刚将小女孩从洪水中救上木板的男子又遇到一位老人。木板太过脆弱，而旁边漂来一段浮木。为了小女孩的生命安全，男子对即将溺水的老人采取了一定的措施。最终小女孩得救，老人死亡。男子采取的措施是……	救上	浮木
10	Doing harm	某病重的妇人手术失败，病痛的折磨让她不堪忍受。为了减轻妇人的痛苦，丈夫采取了一定的措施。最终妇人死亡。丈夫采取的措施是……	闷死	妇人
	Allowing harm	某病重的妇人手术失败，即使再次手术也无济于事。为了减轻妇人的痛苦，丈夫采取了一定的措施。最终妇人死亡。丈夫采取的措施是……	放弃	治疗
	No harm	某病重的妇人手术失败，导致妇人内心非常的绝望。为了减轻妇人的痛苦，丈夫采取了一定的措施。最终妇人死亡。丈夫采取的措施是……	陪伴	妇人
11	Doing harm	某士兵在执行秘密任务时中了敌军陷阱。敌军一定会对他严刑拷打获取信息。为了更多人的安全，队长对该士兵采取了一定的措施。最终其他人脱险，该士兵死亡。队长采取的措施是……	刺死	士兵
	Allowing harm	某士兵在执行秘密任务时中了敌军陷阱。没有足够的时间对该士兵进行营救。为了更多人的安全，队长对该士兵采取了一定的措施。最终其他人脱险，该士兵死亡。队长采取的措施是……	放弃	士兵

	No harm	某士兵在执行秘密任务时中了敌军陷阱。队长了解一些解除这种机关的知识。为了更多人的安全，队长对该士兵采取了一定的措施。最终其他人脱险，该士兵死亡。队长采取的措施是……	救援	士兵
12	Doing harm	深夜某医药实验室毒气泄漏后自动排入一间双人病房。癌症老人戴着氧气面罩而年轻病人没有。为了挽救年轻病人的生命，值班员对癌症老人采取了一定的措施。最终年轻人获救，癌症老人死亡。值班员对癌症老人采取的措施是……	夺走	面罩
	Allowing harm	深夜某医药实验室毒气泄漏后自动排入一间双人病房。时间紧急而值班员无法同时救两位病人。为了挽救年轻病人的生命，值班员对癌症老人采取了一定的措施。最终年轻人获救，癌症老人死亡。值班员对癌症老人采取的措施是……	放弃	老人
	No harm	深夜某医药实验室毒气泄漏后自动排入一间双人病房。值班员无法同时救走两人而同事恰好过来接班。为了拯救两位病人的生命，值班员对同事采取了一定的措施。最终年轻病人获救，癌症老人死亡。值班员对同事采取的措施是……	请求	协助
13	Doing harm	警方抓获了在人口密集区安装了定时炸弹的犯罪分子。此时罪犯的妻子正在旁边。为了逼罪犯尽快交代炸弹藏匿地点，某警官对罪犯怀孕的妻子采取了一定的措施。最终警方及时找到了炸弹，罪犯的妻子生命垂危。该警官采取的措施是……	殴打	孕妇
	Allowing harm	警方抓获了在人口密集区安装了定时炸弹的犯罪分子。此时罪犯的妻子突然早产。为了逼罪犯尽快交代炸弹藏匿地点，某警官对罪犯早产的妻子采取了一定的措施。最终警方及时找到了炸弹，罪犯的妻子生命垂危。该警官采取的措施是……	不顾	孕妇
	No harm	警方抓获了在人口密集区安装了定时炸弹的犯罪分子。此时罪犯的妻子突然早产。为了感化罪犯并交代炸弹藏匿地点，某警官对罪犯早产的妻子采取了一定的措施。最终警方及时找到了炸弹，罪犯的妻子生命垂危。该警官采取的措施是……	救助	孕妇
14	Doing harm	警方抓获了在人口密集区安装了定时炸弹的犯罪分子。此时罪犯的的小女儿正在旁边。为了逼罪犯交代炸弹的藏匿地点，某警官对罪犯的小女儿采取了一定的措施。最终警方及时排除炸弹，罪犯的小女儿生命垂危。该警官采取的措施是……	毒打	女孩
	Allowing harm	警方抓获了在人口密集区安装了定时炸弹的犯罪分子。此时罪犯的的小女儿哮喘发作。为了逼罪犯交代炸弹的藏匿地点，某警官对哮喘的女孩儿采取了一定的措施。	不顾	女孩

		最终警方及时排除炸弹，哮喘发作的女孩生命垂危。该警官采取的措施是……		
	No harm	警方抓获了在人口密集区安装了定时炸弹的犯罪分子。此时罪犯的的小女儿哮喘发作。为了让罪犯交代炸弹的藏匿地点，某警官对哮喘的女孩儿采取了一定的措施。最终警方及时排除炸弹，哮喘发作的女孩儿生命垂危。该警官采取的措施是……	救助	女孩
15	Doing harm	台风过后有树干斜倒在动物园老虎圈的围墙上。饲养员必须尽快移开树干，防止老虎顺着树干爬到圈外，威胁众多游客的生命安全。此时有一名游客正站在虎圈旁边。为争取时间移开树干，饲养员对该游客采取了一定的措施。最终树干被及时移开，该游客被老虎咬死。饲养员采取的措施是……	推下	游客
	Allowing harm	台风过后有树干斜倒在动物园老虎圈的围墙上。饲养员必须尽快移开树干，防止老虎顺着树干爬到圈外，威胁众多游客的生命安全。此时有一名游客意外掉入虎圈内。为抓紧时间移开树干，饲养员对该游客采取了一定的措施。最终树干被及时移开，该游客被老虎咬死。饲养员采取的措施是……	不顾	游客
	No harm	台风过后有树干斜倒在动物园老虎圈的围墙上。饲养员必须尽快移开树干，防止老虎顺着树干爬到圈外，威胁众多游客的生命安全。此时有一名游客执意要靠近虎圈。为顾全该游客的安全，饲养员对该游客采取了一定的措施。最终树干被及时移开，该游客被老虎咬死。饲养员采取的措施是……	警告	游客
16	Doing harm	动物园的装修工不慎将木材斜倒在狮子馆的围墙上。饲养员必须尽快移开木材，防止狮子顺着木材爬到圈外攻击游客。此时狮圈旁边站着一位游客。为引开狮子以移走木材，饲养员对该游客采取了一定的措施。最终木材被及时移开，该游客被狮子咬死。饲养员采取的措施是……	推下	游客
	Allowing harm	动物园的装修工不慎将木材斜倒在狮子馆的围墙上。饲养员必须尽快移开木材，防止狮子顺着木材爬到圈外攻击游客。此时一名游客不慎掉入狮圈。为了能够及时移走木材，饲养员对该游客采取了一定的措施。最终木材被及时移开，该游客被狮子咬死。饲养员采取的措施是……	不顾	游客
	No harm	动物园的装修工不慎将木材斜倒在狮子馆的围墙上。饲养员必须尽快移开木材，防止狮子顺着木材爬到圈外攻击游客。此时一名游客执意靠近狮圈。为了顾全该游客	警告	游客

		的安全，饲养员对该游客采取了一定的措施。最终树干被及时移开，该游客被狮子咬死。饲养员采取的措施是……		
17	Doing harm	某轮船触礁后船长带领乘客们逃往救生船。最后一名大胖子上来后，救生船超载下沉。为了挽救更多乘客的生命，船长对大胖子采取了一定的措施。最终多数人得救，大胖子淹死。船长采取的措施是……	推下	胖子
	Allowing harm	某轮船触礁后船长带领乘客们逃往救生船。最后一名大胖子要上来时，已经没有空间。为了挽救更多乘客的生命，船长对大胖子采取了一定的措施。最终多数人得救，大胖子淹死。船长采取的措施是……	放弃	胖子
	No harm	某轮船触礁后船长带领乘客们逃往救生船。一名病重的大胖子上来时，船上还有空间。为了挽救更多乘客的生命，船长对大胖子采取了一定的措施。最终多数人得救，胖子死亡。船长采取的措施是……	扶上	小船
18	Doing harm	船上的救生员发现一条鲨鱼正游向在海水中嬉戏的五名游客。此时船上的一名青年不慎划伤流血。为了引开鲨鱼，挽救更多的生命，救生员对流血的青年采取了一定的措施。最终五名游客获救，该青年被鲨鱼吞食。救生员采取的措施是……	推下	青年
	Allowing harm	船上的救生员发现一条鲨鱼正游向在海水中嬉戏的五名游客。此时船上的一名青年不慎掉入海中。为了争取时间，挽救更多的生命，救生员对流血的青年采取了一定的措施。最终五名游客获救，该青年被鲨鱼吞食。救生员采取的措施是……	不顾	青年
	No harm	船上的救生员发现一条鲨鱼正游向在海水中嬉戏的五名游客。此时船上一名青年正拿着可能会引来鲨鱼的新鲜鸡肉。为了挽救游客，并顾全青年安全，救生员取了一定的措施。最终五名游客获救，该青年被鲨鱼吞食。救生员采取的措施是……	提醒	青年
19	Doing harm	海啸中幸存的师生们遇到一名重伤的男子。男子攀上小船求助。为了赶快把孩子们送到安全的地方，老师采取了一定的措施。最终孩子们获救，受伤男子死亡。老师采取的措施是……	推下	男子
	Allowing harm	海啸中幸存的师生们遇到一名昏迷的男子。老师已无力救助他人。为了赶快把孩子们送到安全的地方，老师采取了一定的措施。最终孩子们获救，受伤男子死亡。老师采取的措施是……	放弃	男子

	No harm	海啸中幸存的师生们遇到一名重伤的男子。船上还剩余一些空间。为了赶快把更多人送到安全的地方，老师采取了一定的措施。最终孩子们获救，受伤男子死亡。老师采取的措施是……	救起	男子
20	Doing harm	某探险队因触犯孤岛上土著人的禁忌而被追杀。必须献出一人用来祭祀才能让他们平息愤怒。为了使更多人获救，队长对重伤的翻译采取了一定的措施。最终其他人成功逃生，翻译死亡。队长采取的措施是……	交出	翻译
	Allowing harm	某探险队因触犯孤岛上土著人的禁忌而被追杀。必须献出一人用来祭祀才能让他们平息愤怒。为了使更多人获救，队长对掉队的翻译采取了一定的措施。最终其他人成功逃生，翻译死亡。队长采取的措施是……	放弃	翻译
	No harm	某探险队因触犯孤岛上土著人的禁忌而被追杀。必须献出一人用来祭祀才能让他们平息愤怒。为了使更多人获救，队长对重伤的翻译采取了一定的措施。最终其他人成功逃生，翻译死亡。队长采取的措施是……	背着	翻译
21	Doing harm	某载有数名游客的直升机突发故障即将失事。而直升机上的降落伞却少了一顶。为了使更多的人能够逃生，驾驶员对心脏病发的那名游客采取了一定的措施。最终其他游客得救，该游客死亡。驾驶员 采取的措施是……	夺走	伞包
	Allowing harm	某载有数名游客的直升机突发故障即将失事。没有足够时间为所有人进行跳伞指导。为了使更多的人能够逃生，驾驶员对心脏病发的那名游客采取了一定的措施。最终其他游客得救，该游客死亡。驾驶员采取的措施是……	放弃	指导
	No harm	某载有数名游客的直升机突发故障即将失事。而有一名游客对跳伞极度恐惧。为了使更多的人能够逃生，驾驶员对害怕跳伞的那名游客采取了一定的措施。最终其他游客得救，该游客死亡。驾驶员采取的措施是……	鼓励	游客
22	Doing harm	矿井考察时发生意外坍塌，六名考察队员被困在矿下，氧气罐却即将用尽。为使更多人能够等到救援，队长对重伤昏迷的同伴采取了一定的措施。最终其他人得救，该同伴死亡。队长对该同伴采取的措施是……	夺走	氧气
	Allowing harm	矿井考察时发生意外坍塌，六名考察队员被困在矿下，二次坍塌即将发生。为使更多人能够成功逃生，队长对重伤昏迷的同伴采取了一定的措施。最终其他人得救，	丢下	同伴

		该同伴死亡。队长对该同伴采取的措施是……		
	No harm	矿井考察时发生意外坍塌，六名考察队员被困在矿下，救援队已经在赶来。为使更多人能够等到救援，队长对重伤昏迷的同伴采取了一定的措施。最终其他人得救，该同伴死亡。队长对该同伴采取的措施是……	照料	同伴
23	Doing harm	医生诊断某婴儿患有绝症并会带来极大的病痛。为了不让孩子经受病痛的折磨，母亲采取了一定的措施。最终该婴儿死亡。母亲采取的措施是……	淹死	婴儿
	Allowing harm	医生诊断某婴儿患有绝症，且治疗过程非常痛苦。为了不让孩子经受病痛的折磨，母亲采取了一定的措施。最终该婴儿死亡。母亲采取的措施是……	放弃	治疗
	No harm	医生诊断某婴儿的疾病难以治愈。为了不让孩子经受病痛的折磨，母亲采取了一定的措施。最终该婴儿死亡。母亲采取的措施是……	寻访	名医
24	Doing harm	医生诊断某婴儿患有罕见疾病，无法彻底治愈且十分痛苦。为了不让孩子经受病痛的折磨，父亲采取了一定的措施。最终该婴儿死亡。父亲采取的措施是……	摔死	婴儿
	Allowing harm	医生诊断某婴儿患有罕见疾病，手术成功的机会非常渺茫。为了不让孩子经受病痛的折磨，父亲采取了一定的措施。最终该婴儿死亡。父亲采取的措施是……	放弃	治疗
	No harm	医生诊断某婴儿患有罕见疾病，治愈的机会十分渺茫。为了不让孩子经受病痛的折磨，父亲采取了一定的措施。最终该婴儿死亡。父亲采取的措施是……	寻访	名医
25	Doing harm	在草原中休息的商队遇到了狮子的袭击。已经有一位同伴重伤流血。为了引开狮子，带更多人上车逃生，队长对该同伴采取了一定的措施。最终其他人安全逃离，该同伴死亡。队长采取的措施是……	推下	同伴
	Allowing harm	在草原中休息的商队遇到了狮子的袭击。一位同伴受伤掉下了货车。为了摆脱狮子，及时带更多人逃生，队长对该同伴采取了一定的措施。最终其他人安全逃离，该同伴死亡。队长采取的措施是……	不顾	同伴
	No harm	在草原中休息的商队遇到了狮子的袭击。一位同伴受到攻击掉下货车。为了带更多人逃生，队长对该同伴采取了一定的措施。最终其他人安全逃离，该同伴死亡。队长采取的措施是……	救走	同伴
26	Doing harm	在沙漠中探险的团队遇到了野狼的袭击。已经有一位同伴重伤流血。为了引开野狼，	推下	同伴

		带更多人上车逃生，队长对该同伴采取了一定的措施。最终其他人安全逃离，该同伴死亡。队长采取的措施是……		
	Allowing harm	在沙漠中探险的团队遇到了野狼的袭击。一位同伴受伤掉下了货车。为了摆脱野狼，及时带更多人逃生，队长对该同伴采取了一定的措施。最终其他人安全逃离，该同伴死亡。队长采取的措施是……	不顾	同伴
	No harm	在沙漠中探险的团队遇到了野狼的袭击。某同伴受到攻击掉下货车。为了挽救更多的生命，队长对该同伴采取了一定的措施。最终其他人安全逃离，该同伴死亡。队长采取的措施是……	救助	同伴
27	Doing harm	致命的流行病蔓延后药价飞涨。此时药房又意外起火。为了拿走药品，用来拯救病人，助理对试图逃生的医生采取了一定的措施。最终众多病人得救，医生死亡。助理对医生采取的措施是……	困住	医生
	Allowing harm	致命的流行病蔓延后药品紧缺。此时药房又意外起火。为了抢救药品，用来拯救病人，助理对被困火海的医生采取了一定的措施。最终众多病人得救，医生死亡。助理对医生采取的措施是……	放弃	医生
	No harm	致命的流行病蔓延后药价飞涨。此时药房又意外起火。为了让医生降低药价，拯救病人，助理对被困火海的医生采取了一定的措施。最终众多病人得救，医生死亡。助理对医生采取的措施是……	营救	医生
28	Doing harm	某地质考察小组遇到意外仅靠一条树藤坠在悬崖上。已经有一位队员体力不支。为了防止树藤断裂使所有人丧生，领队对该队友采取了一定的措施。最终其他人脱险，该队友坠崖身亡。领队采取的措施是……	踢下	队友
	Allowing harm	某地质考察小组遇到意外仅靠一条树藤坠在悬崖上。已经有一位队友即将滑下。为了防止树藤断裂使所有人丧生，领队对该队友采取了一定的措施。最终其他人脱险，该队友坠崖身亡。领队采取的措施是……	不顾	队友
	No harm	某地质考察小组遇到意外仅靠一条树藤坠在悬崖上。有一位队友试图爬上悬崖。为了防止树藤断裂使所有人丧生，领队对该队友采取了一定的措施。最终其他人脱险，该队友坠崖身亡。领队采取的措施是……	协助	队友

29	Doing harm	某攀岩小组遇到意外仅靠一条绳索坠在峭壁上。已经有一位队员体力透支。为了防止绳索断裂使所有人丧生，组长对该队友采取了一定的措施。最终其他人脱险，该队友坠落悬崖。组长采取的措施是……	踢下	队友
	Allowing harm	某攀岩小组遇到意外仅靠一条绳索坠在峭壁上。已经有一位队友即将滑下。为了防止绳索断裂使所有人丧生，组长对该队友采取了一定的措施。最终其他人脱险，该队友坠落悬崖。组长采取的措施是……	不顾	队友
	No harm	某攀岩小组遇到意外仅靠一条绳索坠在峭壁上。有一位队友试图爬上峭壁。为了防止树绳索裂使所有人丧生，组长对该队友采取了一定的措施。最终其他人脱险，该队友坠落悬崖。组长采取的措施是……	协助	队友
30	Doing harm	某押送重刑犯的警车意外被困在沙漠中多日。为了挽救严重虚脱的同事的生命，警长采取了一定的措施。最终警长的同事得救，罪犯死亡。警长采取的措施是……	吃掉	罪犯
	Allowing harm	某押送重刑犯的警车意外被困在沙漠中多日。为了带虚脱的同事尽快找到出路，警长采取了一定的措施。最终警长的同事得救，罪犯死亡。警长采取的措施是……	丢下	罪犯
	No harm	某押送重刑犯的警车意外被困在沙漠中多日。为了让罪犯帮忙修理设备以便逃生，警长采取了一定的措施。最终警长和同事得救，罪犯死亡。警长采取的措施是……	解拷	罪犯
31	Doing harm	两名警官在深山里抓捕到罪犯后遭遇了野兽的袭击。同事本身已经负伤，为了引开野兽挽救同事的生命，警长采取了一定的措施。最终警长的同事得救，罪犯死亡。警长采取的措施是……	砍伤	罪犯
	Allowing harm	两名警官在深山里抓捕到罪犯后遭遇了野兽的袭击。同事本身已经负伤，为了能够及时抢救同事的生命，警长采取了一定的措施。最终警长的同事得救，罪犯死亡。警长采取的措施是……	丢下	罪犯
	No harm	两名警官在深山里抓捕到罪犯后遭遇了野兽的袭击。同事本身已经负伤，为了击退野兽挽救同事的生命，警长采取了一定的措施。最终警长的同事得救，罪犯死亡。警长采取的措施是……	解拷	罪犯
32	Doing harm	几名修理盘山公路的工人在泥石流中被困在山洞内多日，完全没有食物。为了挽救更多的生命，队长对失血过多的同伴采取了一定的措施。最终其他人获救，该同伴	吃掉	同伴

		死亡。队长采取的措施是……		
	Allowing harm	几名修理盘山公路的工人在泥石流中被困在山洞内。为了尽快带更多同伴逃离即将坍塌的山洞，队长对失血过多的同伴采取了一定的措施。最终其他人获救，该同伴死亡。队长采取的措施是……	丢下	同伴
	No harm	几名修理盘山公路的工人在泥石流中被困在山洞内。为了带更多同伴离开山洞寻找出路，队长对失血过多的同伴采取了一定的措施。最终其他人获救，该同伴死亡。队长采取的措施是……	背起	同伴
33	Doing harm	几名登山者在山体塌方中被困在悬崖下多日。在完全没有食物的情况下，为了保全多数人的生命，队长对已经奄奄一息的同伴采取了一定的措施。最终其他人幸存，该同伴死亡。队长对该同伴采取的措施是……	吃掉	同伴
	Allowing harm	几名登山者在山体塌方中被困在悬崖下多日。在等不到任何救援的情况下，为了带更多的人逃生，队长对已经奄奄一息的同伴采取了一定的措施。最终其他人幸存，该同伴死亡。队长对该同伴采取的措施是……	丢下	同伴
	No harm	几名登山者在山体塌方中被困在悬崖下多日。好在食物和水源暂时足够，为了保全多数人的生命，队长对已经受了重伤的同伴采取了一定的措施。最终其他人幸存，该同伴死亡。队长采取的措施是……	照料	同伴
34	Doing harm	某观光潜水艇突发严重故障而一位游客突然精神失常，严重影响逃生秩序。为了多数人能够及时逃生，负责人对该游客采取了一定的措施。最终其他游客成功逃生，精神失常的那名游客死亡。负责人采取的措施是……	打晕	游客
	Allowing harm	某观光潜水艇突发严重故障而一位游客突发精神失常，强烈拒绝逃生。为了指导多数人及时逃生，负责人对该游客采取了一定的措施。最终其他游客成功逃生，精神失常的那名游客死亡。负责人采取的措施是……	放弃	游客
	No harm	某观光潜水艇突发严重故障而一位游客突发精神失常，严重影响逃生秩序。为了多数人能够及时逃生，负责人对该游客采取了一定的措施。最终其他游客成功逃生，精神失常的那名游客死亡。负责人采取的措施是……	安抚	游客
35	Doing harm	几名武器专家被恐怖分子严刑逼供。有一位同事已经不堪忍受折磨，即将招供。为	毒死	同事

		了保守机密，组长对该同事采取了一定的措施。最终机密得以保守，该同事死亡。组长采取的措施是……		
	Allowing harm	几名武器专家被恐怖分子严刑逼供。有一位同事已经不堪忍受折磨，决定自杀。为了保守机密，组长对该同事采取了一定的措施。最终机密得以保守，该同事死亡。组长采取的措施是……	任其	自杀
	No harm	几名武器专家被恐怖分子严刑逼供。有一位同事已经不堪忍受折磨，犹豫不决。为了保守机密，组长对该同事采取了一定的措施。最终机密得以保守，该同事死亡。组长采取的措施是……	劝说	同事
36	Doing harm	从斜坡滑下的小型拖拉机冲向正在下坡玩闹的几个小孩。此时有一个大个子盲人正好路过。为了改变拖拉机下滑的方向，挽救孩子们的生命，清洁工对大个子盲人采取了一定的措施。最终孩子们得救，盲人被拖拉机撞死。清洁工采取的措施是……	猛推	盲人
	Allowing harm	从斜坡滑下的小型拖拉机冲向正在下坡玩闹的几个小孩。此时有一个大个子盲人走向拖拉机。为了改变拖拉机下滑的方向，挽救孩子们的生命，清洁工对大个子盲人采取了一定的措施。最终孩子们得救，盲人被拖拉机撞死。清洁工采取的措施是……	不顾	盲人
	No harm	从斜坡滑下的小型拖拉机冲向正在下坡玩闹的几个小孩。此时一名盲人敏锐地发现问题，试图阻止拖拉机下滑。为了拯救更多人的生命，清洁工对大个子盲人采取了一定的措施。最终孩子们得救，盲人被拖拉机撞死。清洁工采取的措施是……	协助	盲人
37	Doing harm	一场大地震后医院药品严重不足，而药商的仇人正好找院长开药。为了拯救更多的生命，院长对该病人采取了一定的措施。最终为医院募得了大批药品，病人死亡。院长采取的措施是……	毒死	病人
	Allowing harm	一场大地震后医院药品严重不足，而药商的仇人正好到该医院手术。为了拯救更多的生命，院长对药商的仇人采取了一定的措施。最终为医院募得了大批药品，病人死亡。院长采取的措施是……	放弃	手术
	No harm	一场大地震后医院药品严重不足，而某药商的仇人正好到该医院参观。为了拯救更多的生命，院长对药商的仇人采取了一定的措施。最终为医院募得了大批药品，病人死亡。院长采取的措施是……	化解	恩怨

38	Doing harm	某身受重伤的军人逃亡到一个小村落疗养，被追兵发现。为防止追兵迁怒并杀害村民，族长对军人采取了一定的措施。最终村民得以安然无恙，军人死亡。族长对军人采取的措施是……	杀死	军人
	Allowing harm	某身受重伤的军人逃亡到一个小村落，而追兵即将赶来。为防止追兵迁怒并杀害村民，族长对军人采取了一定的措施。最终村民得以安然无恙，军人死亡。族长对军人采取的措施是……	放弃	医治
	No harm	某身受重伤的军人逃亡到一个小村落，而追兵即将赶来。为防止追兵迁怒并杀害村民，族长对军人采取了一定的措施。最终村民得以安然无恙，军人死亡。族长对军人采取的措施是……	护送	军人
39	Doing harm	几名工人在一场矿难中被困在矿下。大家都严重虚脱，为了多数人的生命，队长对失血过多的工友采取了一定的措施。最终其他人得救，失血过多的工友死亡。队长对该工友采取的措施是……	吃掉	工友
	Allowing harm	几名工人在一场矿难中被困在矿下。为了赶在下一次坍塌前带更多人逃生，队长对失血过多的工友采取了一定的措施。最终其他人得救，失血过多的工友死亡。队长对该工友采取的措施是……	丢下	工友
	No harm	几名工人在一场矿难中被困在矿下。为了带领更多的工友寻找逃生的出路，队长对失血过多的工友采取了一定的措施。最终其他人得救，失血过多的同伴死亡。队长对该工友采取的措施是……	背起	工友
40	Doing harm	某商人执意要收回原先交给孤儿院使用的房产，并在与院长商谈的过程中突发心脏病，而药瓶不慎坠地。为避免孩子们流离失所，院长采取了一定的措施。最终孩子们得以继续居住，商人死亡。院长采取的措施是……	踢开	药瓶
	Allowing harm	某商人执意要收回原先交给孤儿院使用的房产，并在与院长商谈的过程中突发心脏病。为避免孩子们流离失所，院长对心脏病发的商人采取了一定的措施。最终孩子们得以继续居住，商人死亡。院长采取的措施是……	不顾	商人
	No harm	某商人考虑要收回原先交给孤儿院使用的房产，并在与院长商谈的过程中突发心脏病。为了让商人收回决定，院长对心脏病发的商人采取了一定的措施。最终孩子们	救助	商人

		得以继续居住，商人死亡。院长采取的措施是……		
41	Doing harm	某军事小组在执行秘密任务时遇到了不明敌我阵营的重伤平民。为了避免消息泄露，威胁组员生命安全，队长采取了一定的措施。最终小组成员安全完成任务，平民死亡。队长采取的措施是……	杀死	平民
	Allowing harm	某军事小组在执行秘密任务时遇到了不明敌我阵营的重伤平民。为了及时完成任务，保证组员生命安全，队长采取了一定的措施。最终小组成员安全完成任务，平民死亡。队长采取的措施是……	不顾	平民
	No harm	某军事小组在执行秘密任务时遇到了不明敌我阵营的重伤平民。为了打探当地的消息，促进任务的完成，队长采取了一定的措施。最终小组成员安全完成任务，平民死亡。队长采取的措施是……	救走	平民
42	Doing harm	数名滑雪者遭遇雪崩而直升机难以在茫茫白雪中搜寻幸存者。似乎只有血迹才能引来救援。为使更多人能够获救，领队对受伤昏迷的同伴采取了一定的措施。最终其他人获救，受伤昏迷的同伴死亡。领队采取的措施是……	杀死	同伴
	Allowing harm	数名滑雪者遭遇雪崩而直升机难以在茫茫白雪中搜寻幸存者。大家再也没有体力照顾伤员。为使更多人能够获救，领队对受伤昏迷的同伴采取了一定的措施。最终其他人获救，受伤昏迷的同伴死亡。领队采取的措施是……	放弃	同伴
	No harm	数名滑雪者遭遇雪崩而直升机难以在茫茫白雪中搜寻幸存者。受伤昏迷的同伴难以再坚持。为使更多人能够获救，领队对受伤昏迷的同伴采取了一定的措施。最终其他人获救，受伤昏迷的同伴死亡。领队采取的措施是……	照顾	同伴
43	Doing harm	与世隔绝的村庄遭遇了连年的灾荒，大批村民饿死却无处逃生。为了保护年轻的生命，村长对虚弱的老人采取了一定的措施。最终年轻人熬过了灾荒的年岁，老人们大多死亡。村长采取的措施是……	吃掉	老人
	Allowing harm	地处偏远的村庄遭遇了连年的灾荒，年轻人考虑外出求生却无力带走老人们。为了更多人的存活，村长采取了一定的措施。最终年轻人熬过了灾荒的年岁，老人们大多死亡。村长采取的措施是……	丢下	老人
	No harm	地处偏远的村庄遭遇了连年的灾荒，老人们虚弱却愿意跟随年轻人外出求生。为了	带上	老人

		更多人的存活，村长采取了一定的措施。最终年轻人熬过了灾荒的年岁，老人们大多死亡。村长采取的措施是……		
44	Doing harm	一艘游轮触礁即将沉没而救生船有限。有一名男子试图强行登船，严重扰乱了逃生秩序。为了让更多人有序逃生，船长采取了一定的措施。最终大部分人得到了救援，该男子死亡。船长采取的措施是……	枪杀	男子
	Allowing harm	一艘游轮触礁即将沉没而救生船有限。有一名男子在强行登船的过程中不慎坠海。为了让更多人有序逃生，船长采取了一定的措施。最终大部分人得到了救援，该男子死亡。船长采取的措施是……	不顾	男子
	No harm	一艘游轮触礁即将沉没而救生船有限。有一名男子志愿帮助妇女儿童先行登船。为了让更多人有序逃生，船长采取了一定的措施。最终大部分人得到了救援，该男子死亡。船长采取的措施是……	协助	男子
45	Doing harm	某公交车被身缚炸药的持刀歹徒劫持。若有人能撞倒歹徒，司机则能趁机将他制服。为了顾全所有乘客的安全，司机对身旁的一名男子采取了一定的措施。最终其他乘客成功脱险，该男子生命垂危。司机采取的措施是……	猛推	男子
	Allowing harm	某公交车被身缚炸药的持刀歹徒劫持。到达目的地之后，劫持了一名男子作为人质。为了顾全所有乘客的安全，司机对这位被劫持的男子采取了一定的措施。最终其他乘客成功脱险，该男子生命垂危。司机采取的措施是……	不顾	男子
	No harm	某公交车被身缚炸药的持刀歹徒劫持。一名男子试图制服歹徒。为了顾全所有乘客的安全，司机对该男子采取了一定的措施。最终其他乘客成功脱险，该男子生命垂危。司机采取的措施是……	协助	男子
46	Doing harm	某男子发现儿子是妻子与他人通奸所生，持刀冲到幼儿园寻找儿子。为了保护更多的孩子，老师对小男孩采取了一定的措施。最终其他孩子没有受到伤害，该小男孩被砍成重伤。老师采取的措施是……	交出	男孩
	Allowing harm	某男子发现孩子是妻子与他人通奸所生，持刀到幼儿园带走了儿子。为了保护更多的孩子，老师对小男孩采取了一定的措施。最终其他孩子没有受到伤害，该小男孩被砍成重伤。老师采取的措施是……	放弃	男孩

	No harm	某男子发现孩子是妻子与他人通奸所生，持刀冲到幼儿园。为了保护更多的孩子，老师送走其他孩子后对小男孩采取了一定的措施。最终其他孩子没有受到伤害，该小男孩被砍成重伤。老师采取的措施是……	保护	男孩
47	Doing harm	某橡皮艇在漂流活动中偏离轨道被困在水坝下的急流中。而一名游客的救生背心被冲走。为了挽救更多的生命，导游对患有绝症的游客采取了一定的措施。最终其他人得救，身患绝症的那名游客死亡。导游采取的措施是……	夺走	背心
	Allowing harm	某橡皮艇在漂流活动中偏离轨道被困在水坝下的急流中。而导游无法同时带所有人逃生。为了挽救更多的生命，导游对患有绝症的游客采取了一定的措施。最终其他人得救，身患绝症的那名游客死亡。导游采取的措施是……	放弃	游客
	No harm	某橡皮艇在漂流活动中偏离轨道被困在水坝下的急流中。而一名游客却突然哮喘发作。为了挽救更多的生命，导游对患有哮喘的游客采取了一定的措施。最终其他人得救，身患绝症的那名游客死亡。导游采取的措施是……	救助	游客
48	Doing harm	六个和平主义者在战乱国家考察时被恐怖分子劫持为人质。为了能换取恐怖分子的信任，救出更多人，翻译员对一名重伤的人质采取了一定的措施。最终其他人得救，重伤的那名人质死亡。翻译员采取的措施是……	杀死	人质
	Allowing harm	六个和平主义者在战乱国家考察时被恐怖分子劫持为人质。为了抓住难得的机会，及时救出更多的人，翻译员对一名重伤的人质采取了一定的措施。最终其他人得救，重伤的那名人质死亡。翻译员采取的措施是……	放弃	人质
	No harm	六个和平主义者在战乱国家考察时被恐怖分子劫持为人质。为了让人质们能够坚持到救援到来，翻译员对一名重伤的人质采取了一定的措施。最终其他人得救，重伤的那名人质死亡。翻译员采取的措施是……	保护	人质
49	Doing harm	空难中幸存的小女孩和老人在荒岛上同时疾病发作。药物所剩不多且老人已不堪忍受病痛折磨。为了拯救小女孩，护士对病重的老人采取了一定的措施。最终小女孩得救，老人死亡。护士采取的措施是……	捂死	老人
	Allowing harm	空难中幸存的小女孩和老人在荒岛上同时疾病发作。药物所剩不多且老人还有多处重伤。为了拯救小女孩，护士对病重的老人采取了一定的措施。最终小女孩得救，	放弃	老人

		老人死亡。护士采取的措施是……		
	No harm	空难中幸存的小女孩和老人在荒岛上同时疾病发作。药物所剩不多但岛上有可替代的草药。为了拯救患者们，护士对两名病患采取了一定的措施。最终小女孩得救，老人死亡。护士采取的措施是……	治疗	两人
50	Doing harm	无国界医生在战乱中给一名残疾人输血时又接到一名急需输血的孕妇。为了拯救孕妇和未出生的孩子的生命，医生采取了一定的措施。最终孕妇得救，残疾人死亡。医生对残疾人采取的措施是……	切断	输血
	Allowing harm	无国界医生在战乱中救治一名重伤残疾人时又接到一名急需抢救的孕妇。为了拯救孕妇和未出生的孩子的生命，医生采取了一定的措施。最终孕妇得救，残疾人死亡。医生对残疾人采取的措施是……	放弃	治疗
	No harm	无国界医生在战乱中抢救一名重伤残疾人和一名孕妇时，同事及时赶到。为了拯救孕妇和未出生的孩子的生命，医生采取了一定的措施。最终孕妇得救，残疾人死亡。医生对同事采取的措施是……	请求	协助
51	Doing harm	某老人游泳时被水草缠住，本能地抓住了身边的小孩。老人紧紧地抓着小孩不放手。为了拯救小孩的生命，管理员采取了一定的措施。最终小孩得救，老人死亡。管理员采取的措施是……	打晕	老人
	Allowing harm	某老人游泳时被水草缠住，本能地抓住了身边的小孩。管理员无法同时救起两人。为了拯救小孩的生命，管理员采取了一定的措施。最终小孩得救，老人死亡。管理员采取的措施是……	放弃	老人
	No harm	某老人游泳时被水草缠住，本能地抓住了身边的小孩。管理员立刻发现了这一情况。为了拯救两人的生命，管理员采取了一定的措施。最终小孩得救，老人死亡。管理员采取的措施是……	救起	两人
52	Doing harm	景区的观光车驶过一座木桥时，司机发现木桥即将坍塌。而前方桥面上有一名导游摔倒了难以站起。若此时刹车，整车乘客都将陷入险境。为了更多人的安全，司机采取了一定的措施。最终车上的游客安全，导游死亡。司机采取的措施是……	碾过	导游
	Allowing harm	景区的观光车驶过一座木桥时，司机发现木桥即将坍塌。而前方桥面上有一名正在	不顾	导游

		行走的导游。若此时停车救走导游，整车乘客都将陷入险境。为了更多人的安全，司机对采取了一定的措施。最终车上的游客安全，导游死亡。司机采取的措施是……		
	No harm	景区的观光车驶过一座木桥时，司机发现木桥即将坍塌。而对面正有一名导游将要上桥。为了更多人安全，司机对采取了一定的措施。最终车上的游客安全，导游死亡。司机采取的措施是……	提醒	导游
53	Doing harm	恐怖分子将定时炸弹绑在一名人质身上，威胁其前往市中心，而救援队伍来不及赶到。为了防止炸弹在人流密集处爆炸，某警官采取了一定的措施。最终周围没有人员伤亡，该人质死亡。警官采取的措施是……	引爆	炸弹
	Allowing harm	恐怖分子将定时炸弹绑在一名人质身上，威胁其前往市中心，而救援队伍来不及赶到。为了在爆炸前抓紧时间疏散人流，某警官采取了一定的措施。最终周围没有人员伤亡，该人质死亡。警官采取的措施是……	放弃	人质
	No harm	恐怖分子将定时炸弹绑在一名人质身上，威胁其前往市中心，而救援队伍来不及赶到。为了保护人质和广大市民的安全，某警官采取了一定的措施。最终周围没有人员伤亡，该人质死亡。警官采取的措施是……	解救	人质
54	Doing harm	马戏团的狮子在表演跳火圈时受到惊吓，跳下舞台。此时必须尽快引开狮子的注意力，否则观众非常危险。为了观众们的安全，驯兽员采取了一定的措施。最终观众们安然无恙，助手失去了一条手臂。驯兽员采取的措施是……	推下	助手
	Allowing harm	马戏团的狮子在表演跳火圈时受到惊吓，扑向助手。为了尽快疏散观众，保证众人的安全，驯兽员对助手采取了一定的措施。最终观众们安然无恙，助手了失去一条手臂。驯兽员采取的措施是……	不顾	助手
	No harm	马戏团的狮子在表演跳火圈时受到惊吓，助手试图控制狮子。为了观众和助手的安全，驯兽员对助手采取了一定的措施。最终观众们安然无恙，助手了失去一条手臂。驯兽员采取的措施是……	帮助	助手
55	Doing harm	某逃脱法律制裁的杀人犯因煤气中毒被送往医院。抢救回来以后，医生得知杀手还在预谋伤害其他人。为了避免更多无辜的生命受害，医生对杀手采取了一定的措施。最终挽救了无辜的生命，杀手死亡。医生采取的措施是……	切断	输氧

	Allowing harm	某逃脱法律制裁的杀人犯因煤气中毒被送往医院。抢救开始之前，医生得知杀手还有预谋伤害其他人。为了避免更多无辜的生命受害，医生对杀手采取了一定的措施。最终挽救了无辜的生命，杀手死亡。医生采取的措施是……	放弃	抢救
	No harm	某逃脱法律制裁的杀人犯因煤气中毒被送往抢救。康复阶段期间，医生得知杀手还有预谋伤害其他人。为了避免更多无辜的生命受害，医生对杀手采取了一定的措施。最终挽救了无辜的生命，杀手死亡。医生采取的措施是……	劝说	杀手
56	Doing harm	装运木材的板车从斜坡上冲向下坡几名伐木工人。有一个大胖子刚好经过。为改变板车下滑方向，挽回更多的生命,队长对大胖子采取了一定的措施。最终其他人得救，大胖子被板车撞死。队长采取的措施是……	猛推	胖子
	Allowing harm	装运木材的板车从斜坡上冲向下坡几名伐木工人。低头看手机的大胖子正走向板车。为改变板车下滑方向，挽回更多的生命，队长对大胖子采取了一定的措施。最终其他人得救，大胖子被板车撞死。队长采取的措施是……	不顾	胖子
	No harm	装运木材的板车从斜坡上冲向下坡几名伐木工人。路过的胖子试图阻止板车。为了拯救工友们的生命。队长对大胖子采取了一定的措施。最终其他人得救，大胖子被板车撞死。队长采取的措施是……	协助	胖子
57	Doing harm	一小孩误闯医药研究室，触发了一种致命的毒气。研究室没有多余的防毒面罩。为拯救孩子的生命，负责人对患有绝症的同事采取了一定的措施。最终毒气得到控制，患有绝症的同事死亡。负责人采取的措施是……	夺走	面罩
	Allowing harm	一小孩误闯医药研究室，触发了一种致命的毒气。负责人无法同时救走孩子和晕倒的同事。为拯救孩子的生命，负责人对晕倒的同事采取了一定的措施。最终毒气得到控制，晕倒的同事死亡。负责人采取的措施是……	放弃	同事
	No harm	一小孩误闯医药研究室，触发了一种致命的毒气。必须有人留守进行处理。为拯救更多人的生命，负责人对坚持留守的同事采取了一定的措施。最终毒气得到控制，留守的同事死亡。负责人采取的措施是……	协助	同事
58	Doing harm	无意间目击歹徒杀人的一家四口，被歹徒持刀追赶。若不设法拖延歹徒，则难以逃脱。为了带孩子们翻墙逃生，男子对扭伤脚的妻子采取了一定的措施。最终男子带	推下	妻子

		孩子们脱险，妻子死亡。男子采取的措施是……		
	Allowing harm	无意间目击歹徒杀人的一家四口，被歹徒持刀追赶。若带上扭伤的妻子，则难以逃脱。为了带孩子们翻墙逃生，男子对扭伤脚的妻子采取了一定的措施。最终男子带孩子们脱险，妻子死亡。男子采取的措施是……	丢下	妻子
	No harm	无意间目击歹徒杀人的一家四口，被歹徒持刀追赶。若要摆脱掉歹徒，只能尝试翻墙。为了带孩子们翻墙逃生，男子对正托孩子上墙的妻子采取了一定的措施。最终男子带孩子们脱险，妻子死亡。男子采取的措施是……	协助	妻子
59	Doing harm	居民楼起火，精神失常的老婆婆和她的孙子还在屋内。老人死死抓住孩子，拒绝逃生。为了挽救孩子的生命，邻居采取了一定的措施。最终孩子得救，老人死亡。邻居采取的措施是……	打晕	老人
	Allowing harm	居民楼起火，精神失常的老婆婆和她的孙子还在屋内。邻居实在无力同时就走两人。为了挽救孩子的生命，邻居采取了一定的措施。最终孩子得救，老人死亡。邻居采取的措施是……	放弃	老人
	No harm	居民楼起火，精神失常的老婆婆和她的孙子还在屋内。老人抱着孩子，拒绝逃生。为了挽救两人的生命，邻居采取了一定的措施。最终孩子得救，老人死亡。邻居采取的措施是……	劝说	老人
60	Doing harm	某癌症少女因对病情极度绝望而多次自杀。为了不让孩子继续经受折磨，母亲采取了一定的措施。最终少女死亡。母亲采取的措施是……	毒死	女儿
	Allowing harm	某癌症少女因不堪忍受化疗的折磨多次自杀。为了减轻治疗带来的痛苦，母亲采取了一定的措施。最终少女死亡。母亲采取的措施是……	放弃	治疗
	No harm	某癌症少女因不堪忍受病痛的折磨多次自杀。为了让孩子变得坚强乐观，母亲采取了一定的措施。最终少女死亡。母亲采取的措施是……	鼓励	女儿

Non-moral dilemmas in Chinese			
**No.**	**Scenario**	**Protagonist's action**	**Object of the protagonist's action**
1	今年夏天异常炎热，而商场的电器有很多促销活动。为了提高舒适度，小李采取了一定的措施。最终舒适地度过了夏天。小李采取的措施是……	安装	空调
2	某学生因经常吃麻辣烫而肠胃不适。为了身体健康，该学生采取了一定的措施。最终肠胃有所好转。该学生采取的措施是……	改掉	习惯
3	市场上的猪肉频频涨价而家里人又比较爱吃鸡肉。为了减少家庭开支，主妇采取了一定的措施。最终家庭开支有所减少。主妇采取的措施是……	购买	鸡肉
4	自习教室太过闷热。为了使空气流通，学长采取了一定的措施。最终屋内不再那么闷热。学长采取的措施是……	开窗	通风
5	小孩不爱吃蔬菜。为了保证孩子的营养，母亲采取了一定的措施。最终孩子爱上了吃蔬菜。母亲采取的措施是……	改进	厨艺
6	某员工赶到公司楼下时电梯门刚要关闭。为了不迟到，该员工采取了一定的措施。最终他在规定时间内到达办公室。该员工采取的措施是……	赶上	电梯
7	雨季来临时,到处都是水坑。为了防止鞋子进水，小王采取了一定的措施。最终小王的鞋子不再进水。小王采取的措施是……	准备	鞋套
8	某商场上架了一批飞机模型。为了买到自己心仪的飞机模型，小王采取了一定的措施。最终小王如愿以偿。小王采取的措施是……	存钱	购买
9	老人在超市购买酱油和毛巾时发现身上没带够钱。为了不耽误做晚饭，老人采取了一定的措施。最终顺做完晚饭。老人采取的措施是……	放弃	毛巾
10	小王有急事需要出差。为了保证家里电路安全，他采取了一定的措施。最终家中电路一切安好。小王采取的措施是……	切断	电源
11	某青年被突来的大雨困在公园。为了在电影开场之前赶到，青年采取了一定的措施。最终他及时赶到了电影院。青年采取的措施是……	冒雨	前行

12	实验室的电脑经常出现问题。为了提高科研效率，老师采取了一定的措施。最终科研效率得到提高。老师采取的措施是……	更换	电脑
